# Modulation of Wnt/β-catenin signaling promotes blood-brain barrier phenotype in cultured brain endothelial cells

**DOI:** 10.1038/s41598-019-56075-w

**Published:** 2019-12-23

**Authors:** Marlyn D. Laksitorini, Vinith Yathindranath, Wei Xiong, Sabine Hombach-Klonisch, Donald W. Miller

**Affiliations:** 1 Department of Pharmacology and Theurapetics, Max Rady College of Medicine, University of Manitoba, Winnipeg, R3E 0T6 Canada; 2 Kleysen Institute of Advanced Medicine, Health Sciences Center, Winnipeg, Manitoba R3E 0T6 Canada; 3 Department of Human Anatomy and Cell Science, Max Rady College of Medicine, University of Manitoba, Winnipeg, R3E 0J9 Canada; 4 Department of Pharmaceutics, Faculty of Pharmacy, Gadjah Mada University, Yogyakarta, 55281 Indonesia

**Keywords:** Molecular neuroscience, Blood-brain barrier

## Abstract

Wnt/β-catenin signaling is important for blood-brain barrier (BBB) development and is implicated in BBB breakdown under various pathophysiological conditions. In the present study, a comprehensive characterization of the relevant genes, transport and permeability processes influenced by both the autocrine and external activation of Wnt signaling in human brain endothelial cells was examined using hCMEC/D3 culture model. The hCMEC/D3 expressed a full complement of Wnt ligands and receptors. Preventing Wnt ligand release from hCMEC/D3 produced minimal changes in brain endothelial function, while inhibition of intrinsic/autocrine Wnt/β-catenin activity through blocking β-catenin binding to Wnt transcription factor caused more modest changes. In contrast, activation of Wnt signaling using exogenous Wnt ligand (Wnt3a) or LiCl (GSK3 inhibitor) improved the BBB phenotypes of the hCMEC/D3 culture model, resulting in reduced paracellular permeability, and increased P-glycoprotein (P-gp) and breast cancer resistance associated protein (BCRP) efflux transporter activity. Further, Wnt3a reduced plasmalemma vesicle associated protein (PLVAP) and vesicular transport activity in hCMEC/D3. Our data suggest that this *in vitro* model of the BBB has a more robust response to exogenous activation of Wnt/β-catenin signaling compared to autocrine activation, suggesting that BBB regulation may be more dependent on external activation of Wnt signaling within the brain microvasculature.

## Introduction

Brain microvessel endothelial cells are the cellular interface separating the blood and its constituents from brain extracellular fluid. Together with astrocytic foot processes, pericytes, and basal lamina, the brain endothelial cells form the blood-brain barrier (BBB). Brain microvessel endothelial cells are characterized by the presence of tight junction proteins, an assortment of uptake and efflux transporters and reduced vesicular transport processes^1–5^. Together these properties allow selective passage of molecules into and out of the brain and thus provides the proper microenvironment to support brain function^6^.

Activation of the Wnt/β-catenin pathway promotes the formation of tight junction proteins and transporters in the developing brain capillaries^7–9^. Dysregulation of Wnt/β-catenin pathways has also been implicated in various CNS disorders that involve BBB breakdown including multiple sclerosis^10^, stroke^11^, Alzheimer’s disease^12^, Huntington’s disease^13^ and brain tumors^14^. Modulating Wnt signaling pathways is of interest therapeutically, however as a target, Wnt remains challenging due to the complexity of the pathways and its multiple players. There are two different signaling pathways that Wnt can activate. The first is the canonical Wnt pathway, also known as the Wnt/β-catenin signaling pathway. The second is the non-canonical Wnt pathway that is divided into a Wnt/planar cell polarity and Wnt/Ca^2+^ utilization pathway^15^. The complexity scales up as the proteins involved in Wnt/β-catenin signaling have multiple isoforms. For example, there are ten Wnt receptors (Frizzled 1–10), four Wnt co-receptors (LRP5, LRP6, ROR2 and RYK), nineteen Wnt ligands (Wnt 1–16) and ten Wnt modulator peptides (DKK, sFRP, and WIF)^16^. Little information is available regarding the distribution, function, and action of each isoform in the brain microvasculature.

Wnt canonical signaling involves binding of Wnt ligand to the Frizzled (Fzd) receptor and co-receptor LRP5/6. Upon binding, the cytoplasmic tail of LRP5/6 is phosphorylated. Binding of Wnt ligand initiates docking of Dishelleved (Dvl) to Fzd and further recruitment of the β-catenin destruction complex (Axin, CK-1, GSK3, APC) to the plasma membrane. In the inactive state, the β-catenin destruction complex is located in the cytosol where it can efficiently process β-catenin for proteosomal degradation. Recruitment of β-catenin destruction complex to the plasma membrane upon Wnt activation leads to a stabilization of β-catenin. The increased levels of β-catenin in the cytosol results in its translocation to the nucleus where it acts as to modulate Wnt/β-catenin target gene transcription^17–20^.

While previous studies have established the role of Wnt/β-catenin signaling in BBB development^7,9,11,21,22^, less is known regarding the role of Wnt in the maintenance of BBB integrity in mature animals as well as Wnt/β-catenin activity in human BBB. The immortalized human brain microvessel cell line, hCMEC/D3, is a commonly used human *in vitro* BBB model^23–28^. Using hCMEC/D3, several laboratories have determined that Wnt/β-catenin signaling regulates P-glycoprotein (Pgp) expression^29,30^. However, comprehensive characterization of the extent that Wnt/β-catenin influences the barrier properties of the hCMEC/D3 model, beyond changing of Pgp drug efflux, has not been reported. In the present studies, the expression profile of Wnt components including Wnt ligands, receptors, co-receptors and modulators were characterized. The studies dissected the contribution of endogenous Wnt ligands released from hCMEC/D3 in establishment of BBB phenotype and compared the alteration in the BBB phenotype of hCMEC/D3 following activation through natural Wnt ligands and downstream kinase inhibition. While hCMEC/D3 produced Wnt ligand, the autocrine Wnt/β-catenin signaling contribution toward brain endothelial barrier function in the present study was minimal. In contrast, hCMEC/D3 were more responsive both in term of expression of genes known to contribute to BBB phenotypes, as well as functional barrier properties, following to exogenous activation of Wnt/β-catenin signaling through natural Wnt ligand or the inhibition of GSK activity. The studies suggest that autocrine activation of Wnt/β-catenin activation in the cerebral vasculature alone is insufficient to induce BBB phenotype. However, activation of Wnt/β-catenin through pharmacological means such as ligand stimulation or modulation of downstream elements in the signaling pathway can impact on the barrier properties of these cells.

## Result

### Expression of Wnt receptors, ligands and modulators in hCMEC/D3

**Figure 1:**
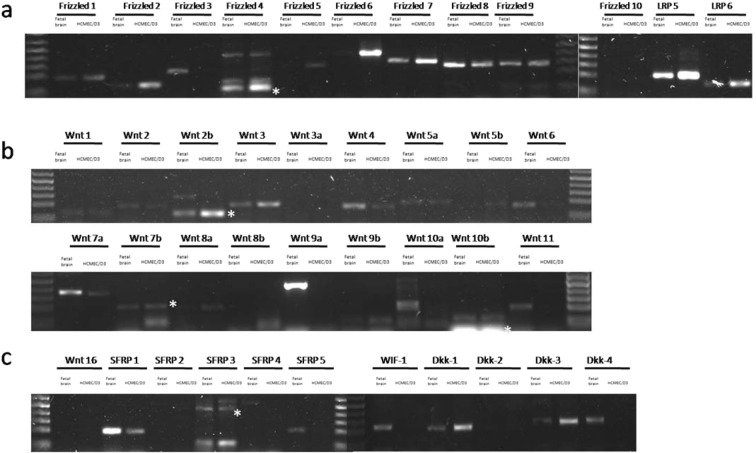
Expression of Wnt/β-catenin components in hCMEC/D3 cells. Expression of Wnt receptors and co-receptors (Panel a), Wnt ligands (Panel b) and Wnt modulators (Panel c) were examined by RT-PCR in confluent hCMEC/D3 monolayers. Total RNA were isolated for further PCR studies. Human Fetal Brain RNA were used as a control positive. Asterisk (*) is the correct PCR product in the primer that shown multiple bands.

Using PCR and qPCR, the various Wnt receptors, activators and modulators were profiled in hCMEC/D3 monolayers. As depicted in Fig. 1, hCMEC/D3 expressed not only Wnt receptors and co-receptors but also several Wnt ligands and Wnt modulators. For the Wnt receptors, Frizzled 3 and Frizzled 10 were undetectable while the other eight Frizzled isoforms were expressed (Fig. 1a). Analysis using qPCR showed a relatively similar expression level among the expressed frizzled receptors (see Supplementary Fig. S1a). However, Frizzled 2 and Frizzled 6 were slightly more abundant compared to the other Frizzled receptors. LRP-5 and LRP-6 were also expressed in the hCMEC/D3 functioning as co-receptors for Wnt/β-catenin signaling (Fig. 1a).

Using the same method, the 19 Wnt ligands were also profiled. As depicted in Fig. 1b, Wnt2b and Wnt3 were the most abundant endogenous canonical Wnt ligands expressed in hCMEC/D3. In addition to Wnt2b and Wnt3, hCMEC/D3 expressed lower levels of the canonical Wnt ligands, Wnt7a, Wnt7b, Wnt6 and Wnt10a. Non canonical Wnt ligands Wnt4, Wnt5a and Wnt5b were also expressed although in reduced amounts compared to Wnt2b and Wnt3 (Fig. 1b). Expression of Wnt3a was not detected in hCMEC/D3. Expression of several Wnt modulators were also identified such as Dkk-1, Dkk-3, SFRP-1 and SFRP-3 (Fig. 1c). Quantification using qPCR showed significantly lower CT number for Dkk-1 and Dkk-3 compared to Wnt2b and Wnt3 suggesting less expression of Wnt ligand compared to Wnt modulator (see Supplementary Fig. S1a). In addition, Dkk-2, Dkk-4, SFRP-2, SFRP-4 and SFRP-5 were not detected under the current experimental conditions.

Proteomics profiling of hCMEC/D3 lysates using SOMAscan assay confirmed the expression of Wnt7a, Dkk-1, Dkk-3, SFRP-1 and SFRP-3 proteins (see Supplementary Fig. S1b). Data analysis from proteomic profiling also indicated expression of RSPO-2, RSPO-3 and RSPO-4 in hCMEC/D3 monolayers (see Supplementary Fig. S1b). The R-spondin (RSPO) are Wnt agonists that have been shown to enhance and potentiate the strength of Wnt/β-catenin activity by preventing frizzled receptor internalization^31–33^.

### Intrinsic (autocrine) Wnt/β-catenin signaling in hCMEC/D3 cells

#### Effects of WntC59 on gene expression and barrier properties

**Figure 2:**
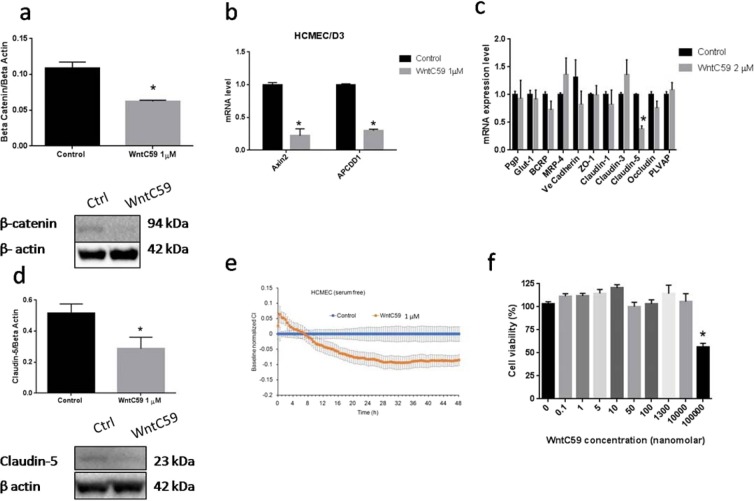
Evidence for autocrine activation of Wnt/β-catenin signaling in hCMEC/D3 monolayers. WntC59 1 µM treatment for 15–20 hour reduced β-catenin abundance (Panel a), downregulated Axin-2 and APCDD1 mRNA expression (Panel b), diminished Claudin-5 mRNA expression (Panel c) and reduced Claudin-5 protein expression (Panel d). The decreased Claudin-5 expression associated with reductions in paracellular barrier were examined using real time cell analyzer (Panel e). hCMEC/D3 cell viability post 24 hours exposure to various concentrations of WntC59 (Panel f). Panel a and d show representative Western blotting of each protein. The cropped blot are used in the figure and full length blot are available at Supplementary Figs. S5 and S6. a and d One tail t-test; n: 3–4. (**b,c**) multitple t-test n: 4. (**e**): n of 4. (**f**) n of 8, One way ANOVA followed by LSD Fisher’s test. (**a**–**f**) *p < 0.05. All values represent the mean ± SEM except (panel e): value is mean ± SD.

**Figure 3:**
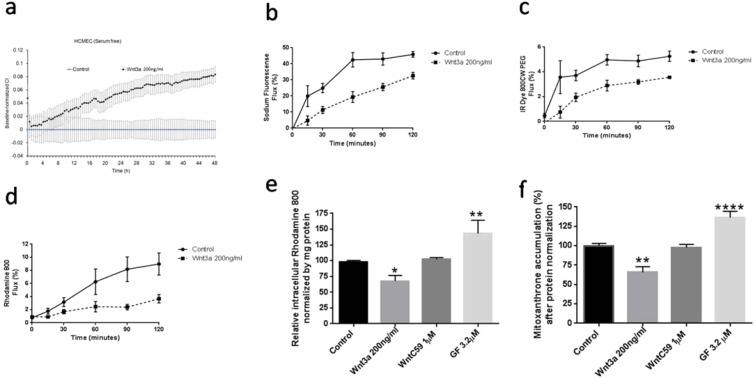
Functional impact of Wnt/β-catenin pathway modulation in hCMEC/D3 monolayers. Effects of Wnt3a exposure on electrical impedance (Panel a); transcellular permeability to small hydrophilic probe sodium fluorescein (Panel b), large hydrophilic probe IRdye 800 PEG (Panel c) and P-glycoprotein permeability probe, Rhodamine 800 (Panel d); and intracellular accumulation of P-glycoprotein and Breast Cancer Resistance Protein dependent fluorescent dyes (Panel e and f, respectively). Electrical impedance changes in response to Wnt3a were measured in real time and expressed as Cell index (CI). Influence of Wnt/β-catenin on transcellular permeability and transport were examined 15-hours following exposure to Wnt3a or WntC59. Values represent the mean ± SEM of 3–4 monolayers for the impendance and permeability studies and 8 monolayers for transporter studies. *p < 0.05; **p < 0.01; ***p < 0.001 as determined using one way ANOVA and LSD Fisher’s test.

With evidence for the expression of multiple Wnt proteins in the cell culture model of the BBB, the contribution of autocrine Wnt activation in the endothelial cells in establishing the BBB phenotype in hCMEC/D3 monolayers was examined. Treatment of cells with WntC59, an inhibitor of Wnt palmitoylation, an essential step for the secretion of Wnt proteins from cells^34,35^, resulted in a 40% reduction in β-catenin protein expression compared to control (p < 0.05, Fig. 2a). Furthermore, WntC59 treatment resulted in reductions in Axin-2 mRNA expression by ~80% (Fig. 2b), indicative of reduced Wnt/β-catenin signaling in the hCMEC/D3 cells. However, examination of β-catenin responsive genes important in establishing the BBB phenotype showed only modest changes in tight junction and adherens junction molecules following WntC59 treatment (Fig. 2c).The most notable change in gene expression following WntC59 treatment was claudin-5 which was reduced approximately 50% at both the mRNA and protein levels compared to vehicle treated controls (p < 0.05, Fig. 2c,d). The reductions in claudin-5 expression observed in the WntC59 treatment group were associated with a decrease in the electrical impedance of hCMEC3/D3 monolayers (Fig. 2e). The reduction in electrical impedance in the WntC59 treatment group was not due to cell toxicity as there was no difference on cell viability as examined by MTT assay (Fig. 2f). As electrical impedance is a surrogate marker for assessing tight junction integrity^36^, the reductions observed in the WntC59 treated cells reflects reduced barrier properties of the cells. Despite the reduction in electrical impedance observed following treatment with WntC59, monolayer permeability assessed using both a small molecular weight marker, sodium fluorescein, and a large molecular weight marker, IR Dye 800CW PEG, did not change significantly between control and WntC59 treatment groups (see Supplementary Fig. S2a,b). Likewise, Pgp and BCRP transporter expression and function was also unaltered in the WntC59-treated hCMEC/D3 cells (Figs. 2c and 3e,f).

#### Effects of ICRT-3 on gene expression and barrier properties

**Figure 4:**
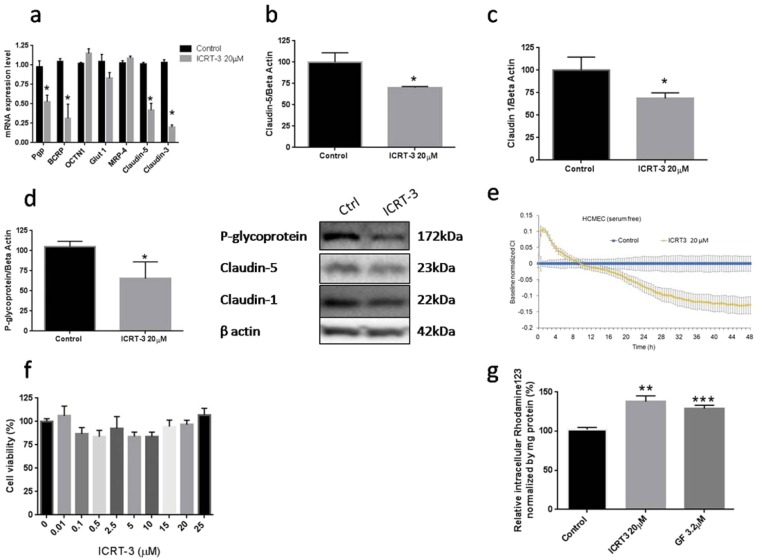
Modulation of Wnt/β-catenin response in hCMEC/D3 using TCF inhibitor ICRT-3. Effects of ICRT-3 treatment on select gene (Panel a) and protein expression (Panels b–d) and endothelial cell function (Panels e and g). Viability of hCMEC/D3 after being exposed to various ICRT-3 concentrations for 24 hours (Panel f). The blots in the middle show representative Westerns for each protein. The cropped blots are used in the figure and full length blots are available in Supplementary Fig. S7. For gene and protein expression and functional studies, cells were exposed to ICRT-3 (20 µM) for 15-hrs. For the electrical impedance studies readings were taken in real-time and expressed as the cell index (CI) normalized to control wells receiving no ICRT-3 (Panel e). Values in all panels represent the mean ± SEM except panel e which is mean ± SD. *p < 0.05 **p < 0.01 ***p < 0.001. Panel a was determined by multiple t-test; (Panel b–d) one tail t-test; (Panel f,g): One way ANOVA followed by LSD Fisher’s test.

In contrast, compared to WntC59, blocking the intrinsic activation of the Wnt/β-catenin in hCMEC/D3 cells at the transcription level using ICRT-3 resulted in more substantial changes in both gene expression and barrier properties. Using ICRT-3, a small molecule inhibitor of β-catenin binding to transcription factor TCF-4^37^, significant reductions in claudin-3 as well as claudin-5, Pgp and BCRP at the mRNA expression level were observed (Fig. 4a). Confirmation of reductions in claudin-5, claudin-1 and Pgp expression were also observed at the protein level using immunoblotting (Fig. 4b–d). The altered expression of adhesion molecules and drug efflux transporters in hCMEC/D3 produced by ICRT-3 treatment was also correlated with changes in the functional properties of the cells. Monolayer electrical impedance measurements in ICRT-3 treated hCMEC/D3 were significantly lower than the control group (Fig. 4e). As the concentrations of ICRT-3 did not change cell viability (Fig. 4f), these reductions in electrical impedance suggests reduced tight junction integrity. Similar to the responses to WntC59, no significant changes in NaF and IR Dye 800CW PEG permeability were detected following ICRT-3 treatment (see Supplementary Fig. S2a,b). P-glycoprotein efflux transporter functional studies using Rhodamine123 showed significant increases in cellular accumulation (~30%; p < 0.01) following ICRT-3 treatment. Such increases were similar in magnitude to those observed following treatment with the Pgp transport inhibitor, GF120918 or GF (Fig. 4g). Together these studies suggest that inhibition of intrinsic Wnt/β-catenin activity with ICRT-3 has minimal impact on paracellular barrier properties, but significantly diminished Pgp transporter function in hCMEC/D3 monolayers.

### Extrinsic activation of Wnt canonical signaling in hCMEC/D3 cells

#### Effects of Wnt3a and LiCl on gene expression and barrier properties

**Figure 5:**
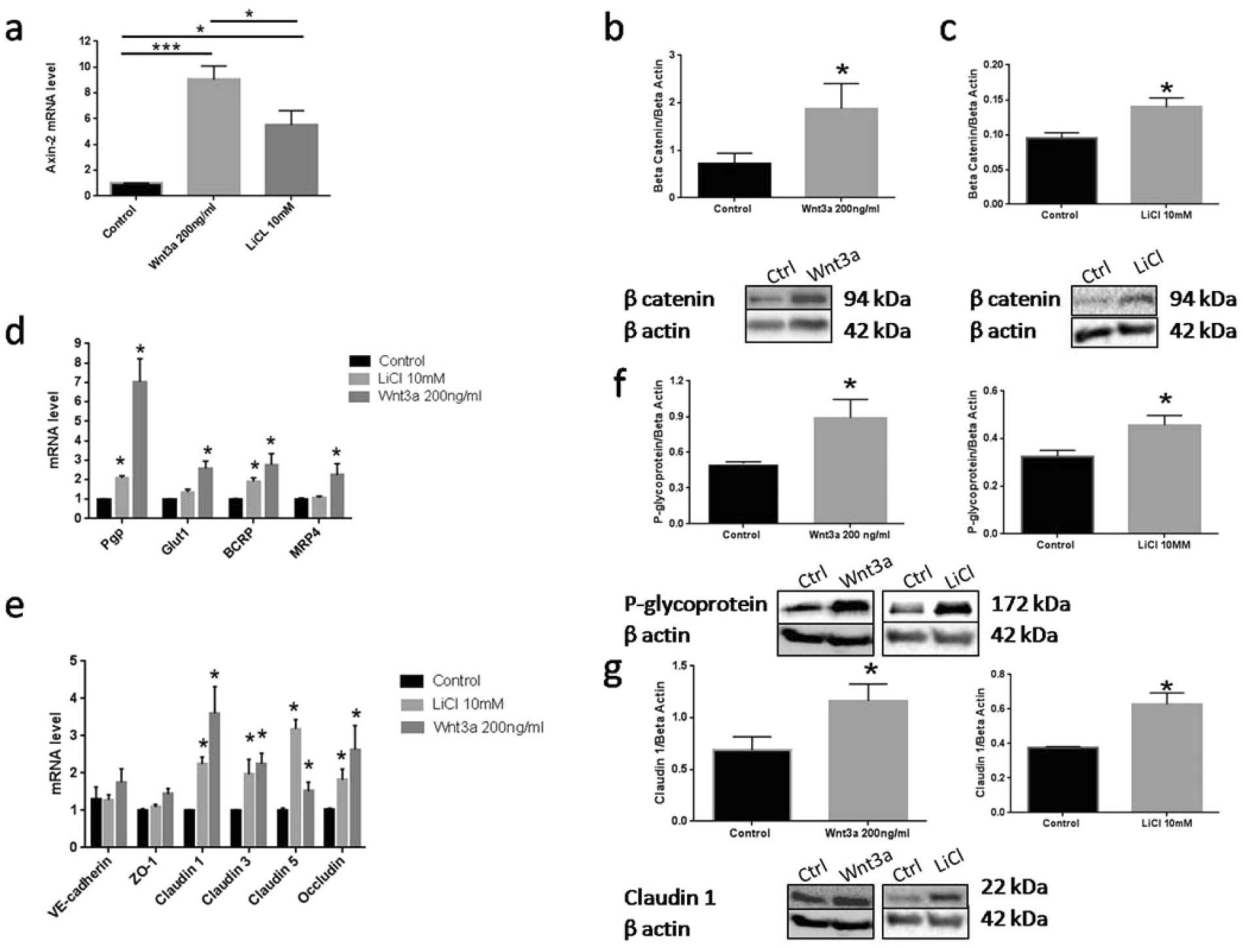
Modulation on Wnt/β-catenin signaling in hCMEC/D3. Natural ligand Wnt3a was a stronger Wnt/β-catenin activator compared to LiCl 10 mM (Panel a). Wnt 3a and LiCl treatment increased β-catenin abundance in whole cell lysate (Panel b,c). The activation of Wnt/β-catenin signaling alters select transporters and intercellular junction proteins (Panel d and e). Wnt3a increased protein expression of Pgp and claudin-1 (Panel f and g). Panels b, c, f, g are representative Western blots of each protein. The cropped blots are used in the figure and full length blots are available in Supplementary Figs. S8–S11: One way ANOVA followed by LSD Fisher’s test.*p < 0.05; ***p < 0.001. (**b**,**c**,**f**,**g**,**h**): One tail t test. P < 0.05. n: 3–4. Mean ± SEM. (**d**,**e**): Two way ANOVA followed by LSD Fisher’s test. n: 4. *p < 0.05.Mean ± SEM.

Studies also examined the extent to which the resulting BBB phenotype of hCMEC/D3 could be altered by exogenous Wnt activators. This was done using Wnt3a, the most potent natural canonical Wnt activator, interacting with a wide range of frizzled receptors^38^, and by treatment with LiCl, a GSK inhibitor that prevents activation of the β-catenin destruction complex. Both Wnt3a and LiCl treatments resulted in β-catenin stabilization and Axin-2 mRNA upregulation. Axin-2, a commonly used marker to identify Wnt/β-catenin activation, was increased 9-fold following Wnt3a treatment while LiCl resulted in 5-fold increase in Axin-2 (Fig. 5a). Although the effects were less robust, both Wnt3a and LiCl increased APCDD1 and cyclin D1, additional target genes for Wnt activation (see Supplementary Fig. S2c). The changes in Axin-2 expression observed with both Wnt3a and LiCl correlated with an increased β-catenin stabilization in the cells (Fig. 5b,c). The β-catenin protein levels were increased by ~2.0 and ~1.5 fold following Wnt3a and LiCl treatment, respectively (Fig. 5b,c).

The impact of Wnt activation on downstream gene expression of intracellular transporters and intercellular junction proteins were examined (Fig. 5d,e). There was a consistent pattern showing that extrinsic activation of Wnt/β-catenin signaling strengthened the BBB phenotype of the hCMEC/D3 cells. The natural ligand, Wnt3a, produced the greatest increase in Axin-2 reporter gene expression and resulted in the highest up-regulation of BBB important transporters such as P-glycoprotein (Pgp), Glucose transporter 1 (Glut-1), breast cancer resistance protein (BCRP), multidrug resistance associated protein 4 (MRP-4) (Fig. 5d). Besides the changes in expression of various transporters important for BBB function, Wnt/β-catenin modulation altered expression of several intercellular junction proteins as well (Fig. 5e). The greatest alterations in BBB gene expression were exhibited by Wnt3a where 7-fold and 3-fold increases in Pgp and claudin-1 mRNA expression, respectively, were observed compared to control monolayers (Fig. 4e, p < 0.05). Up-regulation of Pgp and claudin-1 expression was also observed with LiCl treatment although the magnitude was less than associated with Wnt3a (Fig. 5d,e). In contrast to Wnt3a treated cells, LiCl did not significantly alter Glut-1 or MRP-4 transporter expression in hCMEC/D3 monolayers (Fig. 5d).

The increased expression of BBB relevant transporters and adhesion molecules observed at the mRNA level following extrinsic pharmacological activation of Wnt/β-catenin were also observed at the protein level. Both Wnt3a and LiCl treatments resulted in significant increases in cellular Pgp protein levels by ~2.0 and ~1.6 fold respectively (p < 0.05, Fig. 5f). Similarly, a two-fold increase in claudin-1 protein expression was observed both in Wnt3a and LiCl treatment (p < 0.05, Fig. 5g).

Exposure of hCMEC/D3 to Wnt3a increased monolayer electrical impedance suggesting improvement of the paracellular barrier (Fig. 3a). To identify if the changes in paracellular barrier observed with electrical impedance corresponded to changes in solute permeability, a series of fluorescent markers were used. Treatment with Wnt3a reduced hCMEC/D3 permeabilty to the small molecule diffusion marker NaF (MW 376 g/mol), large molecular weight diffusion marker, IR Dye 800 CW PEG (MW 25–60 kDa) as well as the P-glycoprotein transport substrate, Rhodamine 800 (Fig. 3b–d). As the intracellular levels of NaF and IR Dye 800CW PEG were not influenced by either Wnt3a or LiCl (see Supplementary Fig. S2d,e), the reductions in monolayer permeability observed are likely attributable to reductions in paracellular diffusion of the fluorescent markers. In contrast, as intracellular levels of Rhodamine 800 were reduced following Wnt3a treatment, the decreased permeability of Rhodamine 800 observed following Wnt activation was likely a combination of decreased paracellular leak and enhanced intracellular drug efflux transport (see Supplementary Fig. S2f).

Additional investigation of transporter activity was done using cellular accumulation studies. As depicted in Fig. 3e, the cellular accumulation of the Pgp substrates Rhodamine 800^39^, was decreased by ~20–25% under Wnt3a treatment (p < 0.05). Inhibition of Pgp function by GF increased accumulation of Rhodamine 800 by ~30% (p < 0.01). A similar increase in BCRP drug efflux transporter activity was observed following Wnt3a treatment. Wnt3a reduced mitoxantrone accumulation inside the cell by ~30% (p < 0.05, Fig. 3f). Inhibition of BCRP function by GF120918 (GF) increased mitoxantrone accumulation by ~30% (p < 0.001). Both studies suggested that BCRP and Pgp expression and function was partly regulated by Wnt/β-catenin signaling.

#### Inhibition of LiCl response using ICRT-3

**Figure 6:**
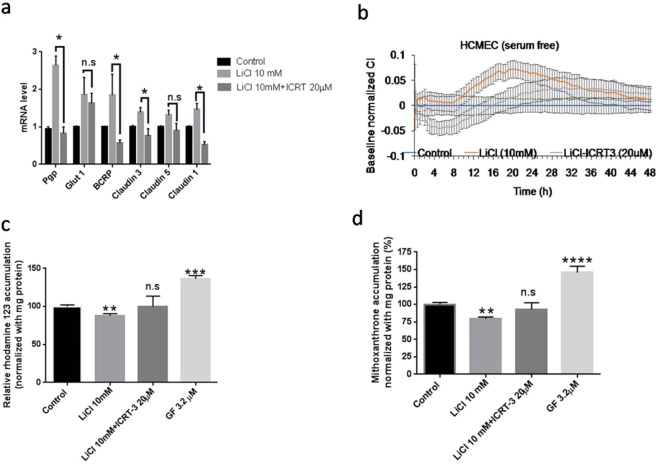
Wnt/β-catenin dependent responses to LiCl treatment in hCMEC/D3 monolayers. ICRT-3 inhibited the effects of LiCl (10 mM) on gene expression (Panel a); paracellular permeability (Panel b) and drug efflux transporter activity (Panels c and d). Values represent the mean ± SEM of n of 4–6 monolayers per treatment group. *p < 0.05; **p < 0.01; ***p < 0.001 as determined using two way ANOVA and LSD Fisher’s test for (Panel a) and one way ANOVA and LSD Fisher’s test for (Panel c,d).

To confirm that the changes in BBB phenotypes observed under LiCl treatment were associated with Wnt/β-catenin activity, a separated set of experiments was done by co-exposure of LiCl and ICRT-3 (Fig. 6). In this studies, combination of LiCl and ICRT-3 prevented the up-regulation of Pgp, BCRP, claudin-1, and claudin-3 mRNA that was previously observed with LiCl treatment (p < 0.05, Fig. 6a). In the electrical impedance studies, ICRT-3 was able to inhibit the improved barrier function initiated by LiCl (Fig. 6b).

Treatment with LiCl reduced accumulation of Rhodamine 123 and mitoxantrone by ~20% in the hCMEC/D3 suggesting improvement of Pgp and BCRP efflux function (p < 0.01, Fig. 6c,d). The effects of LiCl on both Rhodamine 123 and mitoxantrone accumulation were abolished by ICRT-3 (Fig. 6c,d). These studies suggested that improvements in paracellular barrier, P-glycoprotein and BCRP function observed with LiCl treatment were mediated by the binding of β-catenin to TCF-4.

### Alteration of PLVAP expression by Wnt/β-catenin signaling

**Figure 7:**
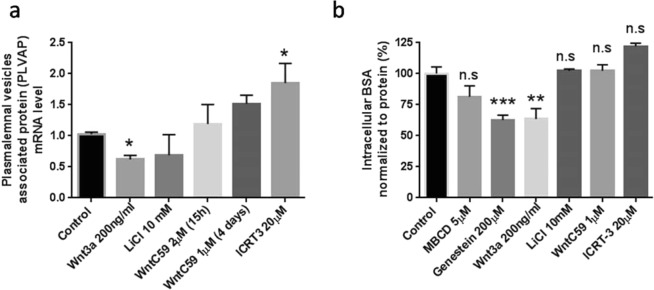
Modulation of Wnt/β-catenin in hCMEC/D3 cells alters endocytic activity. Altered PLVAP gene expression with pharmacological modulation of Wnt activity (Panel a). The reduction of PLVAP gene expression by Wnt3a is associated with less vesicular transport activity (Panel b). a and b, n: 4–6; value was Mean ± SEM. *p < 0.05; **p < 0.005 and ***p < 0.001 determined by One Way ANOVA followed by LSD Fisher’s test

In addition to transporter and intercellular junction proteins, canonical Wnt signaling regulates the expression of plasmalemma vesicle-associated protein (PLVAP/Mecca 32/PV-1). This protein is associated with the ability of the brain endothelial cells to form stomatal caveolae or endothelial fenestrations^40,41^, and increased expression of PLVAP is a marker for BBB dysfunction^42^. Activation of Wnt/β-catenin signaling by Wnt3a reduced PLVAP by ~40% while inhibition of β-catenin activity in nucleus by ICRT-3 increased PLVAP by ~50% (p < 0.05 for both) (Fig. 7a). Functionally, reduction of PLVAP expression under Wnt3a treatment was associated with reduced accumulation of tetramethylrhodamine BSA by ~40% (p < 0.05, Fig. 7b). A similar reduction was observed following treatment with the vesicular transport inhibitor, genestein, (200 µM), suggesting a role of Wnt/β-catenin signaling in regulating vesicular transport in the hCMEC/D3 brain endothelial cell model.

## Discussion

The importance of canonical Wnt signaling for BBB development has been widely studied^7,9,22,43^. Although the Wnt/β-catenin signaling activity in the brain microvasculature is significantly reduced following maturation, its role in BBB maintenance appears vital^8,9^. Evidence in support of this are the studies linking loss of endothelial β-catenin activity in an adult animal model with seizures and the resulting depletion of claudin-1 in the brain microvasculature^11^. Another study observed widespread sulfo-NHS-biotin leakage into the brain upon LRP-5 and LRP-6 conditional knockout in 24-day old mice^22^.

It should be noted that public database analysis (www.genecards.org) confirms that many intracellular transporters and intercellular junction proteins important in maintaining a BBB phenotype have a binding site for TCF4, TCF7 and LEF-1, that are known transcripton factors in the Wnt/β-catenin pathway. Genes for Pgp, BCRP, MRP4, Glut-1, claudin-1, claudin-3 and PLVAP have a regulatory element that interacts with TCF-4. In addition, studies using a TCF4 dominant negative mutant mouse demonstrated reduction in claudin-5 expression^22^. Together these studies suggest that Wnt/β-catenn signaling may play an important role in regulating gene expression important for maintaining the BBB phenotype.

Current understanding suggests that astrocytes are a source for Wnt ligand in the BBB^44^. There is also an emerging evidence to suggest that brain endothelial cells may be activated by Wnt proteins in an autocrine fashion in both *in vivo* and *in vitro* settings^16,45–47^. However, comprehensive studies looking at the functional impact of Wnt/β-catenin activation in human brain endothelial cell either through autocrine and exogenous activation have not been reported. The results of the present study suggest that (1) the contribution of Wnt ligand, produced by brain endothelial cells themselves, towards maintaining the BBB phenotype is likely minimal, (2) modulation of Wnt/β-catenin signaling in brain endothelial cells from exogenous sources can significantly alter BBB function, and (3) the extent of BBB phenotype changes observed with Wnt activators correlated with the magnitude of Wnt/β-catenin produced.

The immortalized human cerebral microvessel endothelial cell line hCMEC/D3 is widely used as an *in-vitro* BBB model due to its ability to retain the brain endothelial phenotype from passage to passage compared human primary brain endothelial cells (HBMEC)^24–28^. While canonical Wnt signaling in the hCMEC/D3 cell model has been reported^29^, the present study is the first to provide a comprehensive profiling of the effects of Wnt/β-catenin signaling, through both endogenous and exogenous activation routes, on the barrier properties of this cell. The current studies in the hCMEC/D3 culture model show the expression of multiple Wnt receptors, with eight frizzled receptors along with LRP-5 and LRP-6. The Wnt receptor profile observed in hCMEC/D3 was similar to previous reports in mouse brain microvessels^7^. Expression of frizzled-4 was particularly high in the hCMEC/D3 BBB culture model. Besides being a receptor for Wnt ligand, frizzled-4 is also important for Norrin, a non traditional ligand for Wnt canonical signaling^48,49^. Endothelial frizzled-4 deletion in the embryonic, postnatal and adulthood is associated with reduction of claudin-5 and increase expression of PLVAP in the cerebellum^22,48^. This suggested the important of Frizzled-4 not only for BBB development but also BBB maintenance.

In addition to Wnt receptors and co-receptors, the hCMEC/D3 also expressed various Wnt ligands including Wnt2b, Wnt3, Wnt4 and Wnt6. The hCMEC/D3 Wnt ligand profiling also showed moderate levels of Wnt7a and Wnt7b, Wnt ligands that were absent in various peripheral endothelial cell preparations^16,45^. In agreement with hCMEC/D3, our RT-PCR examination of primary human brain endothelial cells (HBMECs) also indicated expression of Wnt2b and Wnt3 as the major canonical Wnt ligand (see Supplementary Fig. S3). In general, HBMEC expressed a similar Wnt receptor profile compared to hCMEC/D3 but more limited range of Wnt ligands and Wnt modulators compared to hCMEC/D3. Of note, Wnt7a was expressed in both primary cultured brain endothelial cells as well as hCMEC/D3. Wnt7a has an important role in regulating CNS angiogenesis and Glut 1, PLVAP as well as claudin-5 expression^43,50^. In the mouse embryo, Wnt7a and Wnt7b exhibited the broadest expression pattern in the developing CNS. Wnt7a and Wnt7b double knockout mice were non-viable due to CNS vessel malformation. *In vitro*, Wnt7a treatment was also reported to increase the expression of Glut-1 transporter in mouse brain endothelial cells^7^.

The Wnt profiling of hCMEC/D3 also showed expression of several Wnt modulators including Dkk-1, Dkk-3, SFRP-1 and SFRP-3. Quantitative PCR studies suggested that hCMEC/D3 produced more Wnt modulators compared to Wnt ligands. These findings, together with the increased responses to exogenous Wnt activators versus endogenously released Wnt ligands in the hCMEC/D3, suggest that activation of Wnt/β-catenin in brain endothelial cells is more likely through a paracrine pathway involving Wnt agonists released from neighboring cells such as pericytes, neurons and astrocytes.

Additional evidence in favor of paracrine pathways for activation of Wnt/β-catenin processes in the brain endothelium are the studies using WntC59 to inhibit endogenous release of Wnt from the endothelial cells. Of the various BBB genes examined, only the expression of claudin-5 was significantly reduced following WntC59 treatment. Functionally, although there was a modest decrease in electric impedance of the hCMEC/D3, suggesting changes in tight junction formation, the permeability of both small and large permeability markers was unchanged following WntC59 treatment. Together these findings suggest that the brain endothelial cells have minimal Wnt activation through autocrine routes, but can greatly enhance their barrier properties following exogenous activation through Wnt receptor agonists or downstream pathway modulators.

While these studies showed minimal effect of inhibition of Wnt ligand release on barrier properties of hCMEC/D3, the brain microvessel endothelium has the capability to adjust the level of Wnt activation through autocrine pathways involving Wnt modulators or R-spondin (RSPO). Our studies and others found relatively high expression of RSPO in the human brain endothelial cell at the mRNA and protein level^51^. R-spondin (RSPO) is a non traditional Wnt agonist capable of regulating Wnt/β-signaling strength through altering frizzled receptor turnover^32,52^. Previous studies suggested that brain endothelial cells were the major producer of R-spondin compared to brain pericytes^51^ and addition of RSPO together with Wnt3a increased Wnt/β-catenin activation by 10-fold compared to Wnt3a alone^33^. Thus, any small leak of Wnt release following WntC59 treatment could be potentiated by RSPO and result in some basal activity. In contrast, ICRT-3 blocks the TCF-4 transcription factor. Blocking at the level of the transcription factor would be expected to be more effective way of reducing the intrinsic Wnt activation as the pharmacological intervention occurs downstream of ligand interactions with the membrane receptors. This may explain why the effects observed with ICRT-3 appear to be more robust than the effect observed with WntC59.

While blocking endogenous Wnt ligand release and downstream transcription factor interactions had minimal impact on the BBB properties of the cell culture model, exogenous activation of Wnt/β-catenin using either Wnt3a or LiCl, significantly improved the BBB phenotype in hCMEC/D3 cells. Using Axin-2 expression as a reporter of Wnt/β-catenin activity, the highest activation of Wnt signaling in hCMEC/D3 cells was achieved through its natural ligand (Wnt3a). Activation of canonical Wnt pathways with Wnt3a also produced a robust effects on gene and protein expression involved in the BBB phenotype. This included selected transporters, such as Pgp, Glut-1, BCRP, MRP-4, as well as tight junction proteins including claudin-1, claudin-3, claudin-5 and occludin.

Selection of Wnt3a, from the many Wnt ligands is based on the following. First, Wnt3a is not expressed in our hCMEC/D3 culture model of the BBB, nor in primary human brain microvessel endothelial cells. Studies in other peripheral endothelial cells also suggest that Wnt3a is not a ligand that would be produced by the endothelial cells themselves^16,45^. The source of Wnt3a in the brain is more likely from neuron, astrocytes and pericytes^53–55^. Thus the study with Wnt3a provided us with a way to examine the ability of the brain endothelial cells to respond to non-endothelial based Wnt ligands.

The effects observed with exogenous Wnt3a were more robust compared to the activation of Wnt/β-catenin through GSK inhibition. Similar results have also been observed in the bEnd-3, mouse endothelial cell line where Wnt3a conditioned media improved the BBB phenotype compared to LiCl treatment^21^. This could be explained by high potency of Wnt3a in activating Wnt/β-catenin signaling compared to other canonical Wnt ligands^38^. Unlike Wnt3a which activates the Wnt receptor, and triggers the binding of Axin and prevents the formation of the β-catenin destruction complex, LiCl activates Wnt indirectly through inhibition of GSK3^56^. As only a small pool of GSK3 is associated with Axin, it takes substantial inhibition of GSK3 to impact Wnt/β-catenin signaling. Indeed, it has been reported that GSK3 has to be inhibited by at least 80% before activation of Wnt/β-catenin is observed^57^. Thus there is a limit to activation of Wnt through GSK3 blockers that is not encountered with the Wnt receptor ligands.

In the present studies we also examined the effects of Wnt/β-catenin signaling on plasmalemma vesicle-associated protein PLVAP/Mecca 32 gene expression. This protein is responsible for formation of stomatal fenestrae and caveolae involved in pore formation and vesicular transport, respectively, and is normally expressed at very low levels in brain endothelial cells^40,41^. Treatment with Wnt3a resulted in reductions in both PLVAP expression and fluorescently labeled albumin uptake in hCMEC/D3. As BSA is internalized through a caveoli-mediated process^58^, the reductions in PLVAP expression observed with Wnt3a resulted in reduced endocytosis in the cells. Inhibition of autocrine activation of Wnt signaling with ICRT-3 resulted in an increase in PLVAP gene expression, although no significant alteration in caveoli-mediated endocytosis was observed. These finding are support the previous studies reporting that Wnt/β-catenin signaling influences PLVAP expression in brain endothelial cell^9,22,48,50^.

The hCMEC/D3 monoculture model used in this study allowed examination of the intrinsic canonical Wnt signaling without interference from Wnt ligands produced by other brain cells. In the BBB neurovascular unit, astrocytes and perycites are known to release Wnt ligand for brain endothelial Wnt activity^44,53^. The present studies characterized the impact of Wnt/β-catenin signaling in the hCMEC/D3 cell culture model of the blood brain barrier, demonstrating changes in transporter, paracellular barrier and vesicular endocytosis activity through altered expression of genes important for the BBB phenotype (illustration in Fig. S4). While the present studies showed limited contribution of autocrine Wnt signaling pathways in the hCMEC/D3 to the establishment of the BBB phenotype, a more robust response was observed from exogenous agents that activated the canonical Wnt signaling pathway. The improvement in barrier properties of the hCMEC/D3 following Wnt ligand and LiCl treatment suggests Wnt/β-catenin signaling may ameliorate BBB compromise under various pathophysiological conditions.

## Material and Methods

### Material

hCMEC/D3 were obtained from Dr. Peter Couraud^59^ INSERM, France. WntC59 was purchased from AdooQ Biosciences (Irvine, CA). Human Recombinant Wn3a, and GF120918 (GF) were purchased from R&D Systems (Minneapolis, MN). Rhodamine 800 and Rhodamine 123 were purchased from Sigma (St. Louis, MO). IRDye 800 CW PEG were purchased from LICOR (Lincoln, NB). EBM-2 media was purchased from Lonza (Walkersville, MD). The E-plates for RTCA were obtained from ACEA Biosciences (San Diego, CA). Transwell and tissue culture plates were purchased from Corning (Tewksbury, MA). Trizol and tetramethylrhodamine BSA was purchased from Life Technologies (CA, USA). P-glycoprotein and Claudin-1 antibodies were purchased from Abcam (Cambridge, MA). β-catenin, claudin-5 and β-actin antibodies were purchased from Sigma (St. Louis, MO). Human fetal brain total RNA was purchased from Takara Bio USA, Inc. (Madison, WI). Primers were obtained from Invitrogen (CA, USA) and primer sequence information is provided in Table 1 Supplementary information.

### Cell culture

For cell expansion, hCMEC/D3 were seeded at 10.000 cells/cm^2^ onto T75 flasks (Corning Inc) that had been coated with rat tail collagen 0.1 mg/ml for an hour. Cells were cultured at 37 °C and 5% CO_2_ in EBM-2 media supplemented with 5% FBS, 1% penicillin streptomycin, 1 ng/ml bFGF, 10 mM HEPES, 5 µg/ml ascorbic acid, 1/100 CD lipid concentrate, and 1.4 µM hydrocortisone. Cells were passaged when reaching approximately 80% confluency using 1 ml Trypsin/EDTA. For expression and functional studies, cell were seeded onto culture plates coated with rat tail collagen at a density of 25,000 cells/cm^2^ and used upon reaching confluency (usually within 4–5 days). To insure maintenance of BBB properties the hCMEC/D3 were used at passage 29 to 34^23^.

### Immunoblotting

Cell were solubilized using RIPA buffer (Sigma) supplemented with protease and phosphatase inhibitors (Thermo Scientific). The lysates were subsequently centrifuged at 12.000 rpm for 8 minutes for isolating the whole cell lysate. The protein concentration was determined by Pierce BCA protein analysis (Thermo Scientific). Depending on the protein examined, 20–40 µg of protein was loaded into the SDS-polyacrylamide gel and run for separation at 110 Volt. The proteins were then transferred to PVDF membrane (BioRad) for 2 hour at 200 mAmp. The membrane was subsequently blocked in 5% non skim milk at TBST buffer for an hour at room temperature. Then, the membrane was incubated with the primary antibodies dissolved at 5% non skim milk TBST buffer at 4 °C on the rocking rack. Dilution 1: 1000 were applied for Claudin-1, Claudin-5, P-glycoprotein, β-catenin, GSK3β, phospho-GSK 3β, and phospho-β-catenin. In the next day, membranes were washed thrice with TBST buffer for ten minutes each before being incubated with secondary antibodies for 1 hour at room temperature. Bands were visualized using chemiluminescence at ChemiDoc Imager (Biorad) and analyzed using Image Lab software (Biorad).

### RT PCR

Expression of Wnt receptor, co-receptor, ligand, and modulator were examined in the human cerebral microvessels endothelial cell line (hCMEC/D3) using RT-PCR. Human fetal brain total RNA was used as a positive control for the primer as it expressed most of the Wnt component. Total RNA were isolated using Trizol®. RNA concentration was measured using Nanodrop UV Vis Spectrometer (Fisher Scientific). One microgram of total RNA was converted to cDNA using MLV Reverse Transcriptase enzyme (Invitrogen) with the final volume of 60uL according to the manufacturer’s protocol. 1uL of the cDNA were subjected to PCR using Platinum Taq polymerase (Invitrogen). The cycle was initial denaturation at 94 °C for 2 minutes followed by 30 PCR cycle that consist of 94 °C for 30 seconds for denaturation, 60 °C for 45 second for annealing and 72 °C for 45 second for extension using programmable thermal controller (MJ Research Inc.). Ten microliter of PCR product were eluted in the 1% agarose gel at 104 Volt in 1x TAE buffer. Before the gel solidified, 10 uL of GelGreen (Biotium) were added to the agarose gel for PCR product visualization. The image was visualized using ChemiDoc (BioRad).

### qPCR

RNA isolation was done similar to the previous section. Total RNA was subjected to qPCR using ITaq Universal Syber Green (BioRad) according to manufacturer protocol using QuantStudio 5 (Thermo Fisher).

### P-glycoprotein, BCRP, Glut-1 functional assay

Standard P-glycoprotein functional assay were done at 24 well plate using Rhodamine 123 (2.12 µM) as a Pgp substrate and GF120918 or GF (3.2 µM), as a Pgp inhibitor^60,61^. The cells were treated with Wnt3a, LiCl, WntC59 and ICRT-3 for 15–20 hour before the experiment under serum free media. On the day of experiment, the cells were washed with sterile PBS. Subsequently the cells were equilibrated in 0.5 ml assay buffer for 30 minutes. In this 30 minutes pre-incubation, only the GF group received 3.2 μM GF. The uptake were done for two hour at 37 °C and 5% CO_2_ with 0.5 ml sterile assay buffer (NaCl 150 mM, KCl 4 mM, CaCl_2_ 3.2 mM, MgCl_2_1.2 mM, HEPES 15 mM, Glucose 5 mM and 1% BSA) containing Rhodamine 123 2.12 µM_._ At the end of the experiment, the plates were washed two times with 1 ml of ice cold PBS. To solubilize intercellular Rhodamine 123, an 0.4 ml of 1% Triton X in PBS were added to each well and placed to the −20 °C freezer overnight. Following day, 100 µL of the lysate were transferred to black 96 well plate and were analyzed using Biotek Synergy HT Microplate Reader at excitation 485 nm and emission 528 nm. The amount of intercellular Rhodamine 123 was normalized to protein content using Pierce BCA protein assay and expressed either as nanogram Rhodamine 123 per mg protein or as a percent of the control group.

Alternative P-glycoprotein functional assay were done using Rhodamine 800 0.1 µM as Pgp substrate with GF120918 (GF) 3.2 µM as an inhibitor. The protocol were similar to Rhodamine 123 studies. At the end of the experiment, the intercellular accumulation of Rhodamine 800 was quantified using Odyssey Near Infrared Imager (Licor, Omaha, NE) using 700 nm channel.

BCRP functional studies used mitoxantrone 15 µM as a BCRP substrate and GF120918 (GF) 3.2 µM as a BCRP inhibitor^62^ using similar protocol as Rhodamine 123. Intracellular accumulation of mitoxantrone were quantified using Odyssey Near Infrared Imager (Licor) using 700 nm channel.

Rhodamine 123, Rhodamine 800, and mitoxantrone studies were done in separated sets of experiments. The assay was conducted at 15–20 hour post-treatment with Wnt ligands and modulators.

### Endocytosis activity in hCMEC/D3 cell monolayers

Tetramethylrhodamine conjugated BSA 5 μg/ml was used to study vesicular transport mediated endocytosis^58^. Methyl β-cyclodextrin (MBCD; 0.5 mM) and genestein 200 µM were used as inhibitors of endocytic pathways^63^. Similar protocol as Pgp and BCRP functional studies were used. After uptake for two hours, intercellular accumulation of tetramethyl conjugated BSA was quantified using Biotek Synergy HT Microplate Reader at excitation 530 nm and emission 590 nm

### Permeability studies

Permeability studies were done in the 6 well Transwell 0.4 µm pore polycarbonate membrane after pre-coated with rat tail collagen. hCMEC/D3 were seeded in the density of 25.000 cell/cm^2^. Apical and basolateral chamber were filled with media 1.5 ml and 2.5 ml complete EBM-2 subsequently. The monolayer was used upon reaching confluency, typically 4–5 days after seeding. For the permeability studies, the monolayers were treated with different Wnt activators or inhibitors for 15–20 hours in the absence of serum. On the day of experiment, the media was removed and replaced with assay buffer in both basolateral and apical compartments. The assay buffer in the apical compartment also contained 0.1 µM Rhodamine 800, 0.1 µM IR Dye CW PEG and 1 µM sodium fluorescein to assess monolayer permeability. A hundred microliters samples were removed from the basolateral compartment at various times (0, 15, 30, 60, 90 and 120 minutes) and replaced with equal volume of fresh assay buffer. Samples (10 μl) were also taken from apical compartment at the start and conclusion of permeability study. The samples from the apical and basolateral compartments were placed in black 96-well plates and diluted to 100 μl of assay buffer. Quantitative analysis of the various solutes were performed using an Odyssey Near Infrared Imager (Licor; 700 nm channel for Rhodamine 800 and 800 nm channel for IR Dye 800 CW PEG) and Biotek Synergy HT Microplate Reader at excitation 485 nm and emission 528 nm for sodium fluorescein. The amount of fluorescence activity was quantitated using standard curves for each fluorescent compound. Permeability data were presented as the percent flux.

### Measurement of monolayer electrical impedance

Monolayer electrical impedance were measured using xCELLigence RTCA system. Briefly, the cell was seeded in 16 well E-plate (ACEA Biosciences) that have been coated with rat tail collagen with the density 14,000 cell/well. The E-plate surface was covered with microelectrode that measure the electrical impedance of the monolayer resulting a dimensionless value called Cell Index (CI). The cell was seeded in 200 µL EBM-2 media and been replaced with new media every other day. The impedance figauto-mesurement interval was every 5 minutes. After the impedance reached the plateau for at least 24 hour, the media was removed and replaced with treatment drug with serum free EBM-2. Impedance monitoring was continued 24–48 hour post treatment.

## Supplementary information

SupModulation of Wnt/β-catenin signaling promotes blood-brain barrier phenotype in cultured brain endothelial cells

## Data Availability

The datasets produced during and/or analyzed during the current study can be available from the corresponding author on reasonable requests.
