# Supplementary material for: Modulation of Wnt/β-catenin signaling promotes blood-brain barrier phenotype in cultured brain endothelial cells
Source: Sci Rep. 2019 Dec 23;9:19718. doi: 10.1038/s41598-019-56075-w (PMC6928218; doi:10.1038/s41598-019-56075-w)
Supplement: Supplementary file 1 — SupModulation of Wnt/β-catenin signaling promotes blood-brain barrier phenotype in cultured brain endothelial cells [file 41598_2019_56075_MOESM1_ESM.docx]

**Modulation of Wnt/β-catenin signaling promotes blood-brain barrier phenotype in cultured brain endothelial cells**

Marlyn Laksitorini^1,4^, Vinith Yathindranath^1^, Wei Xiong^1^, Sabine Hombach-Klonisch^3^, Donald W. Miller^1,2^

*^1^Department of Pharmacology and Theurapetics, Max Rady College of Medicine, University of Manitoba, R3E 0T6, Canada.*

*^2.^ Kleysen Institute of Advanced Medicine, Health Sciences Center, Winnipeg, Manitoba, R3E 0T6, Canada.*

*^3.^Department of Human Anatomy and Cell Science, Max Rady College of Medicine, University of Manitoba, R3E 0J9, Canada.*

*^4^Department of Pharmaceutics, Faculty of Pharmacy, Gadjah Mada University, Yogyakarta, 55281, Indonesia.*

**corresponding author: Donald.Miller@umanitoba.ca*

SUPPLEMENTARY INFORMATION


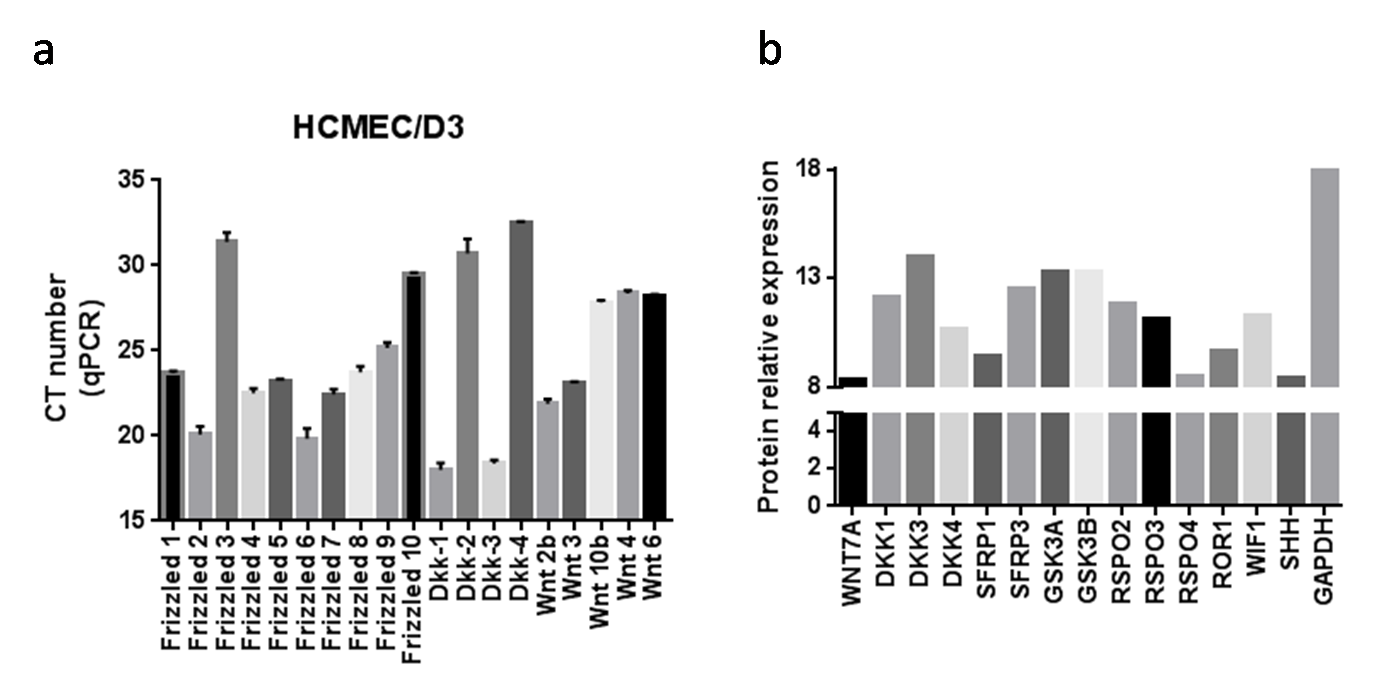
Figure S1: Relative quantification of Wnt component at hCMEC/D3. a) Comparison of major Wnt component expression level at hCMEC/D3 examined using qPCR. RNA sample was isolated from three separated cultured. CT number expressed the abundance of the RNA transcript in the cell. CT number for β-actin was approximately 11.9. b) Relative expression of Wnt component protein expression examined by Somologic Inc. Cell were grown in T75 cell under normal condition until confluence before sent to Somologic Inc for proteomic analysis.


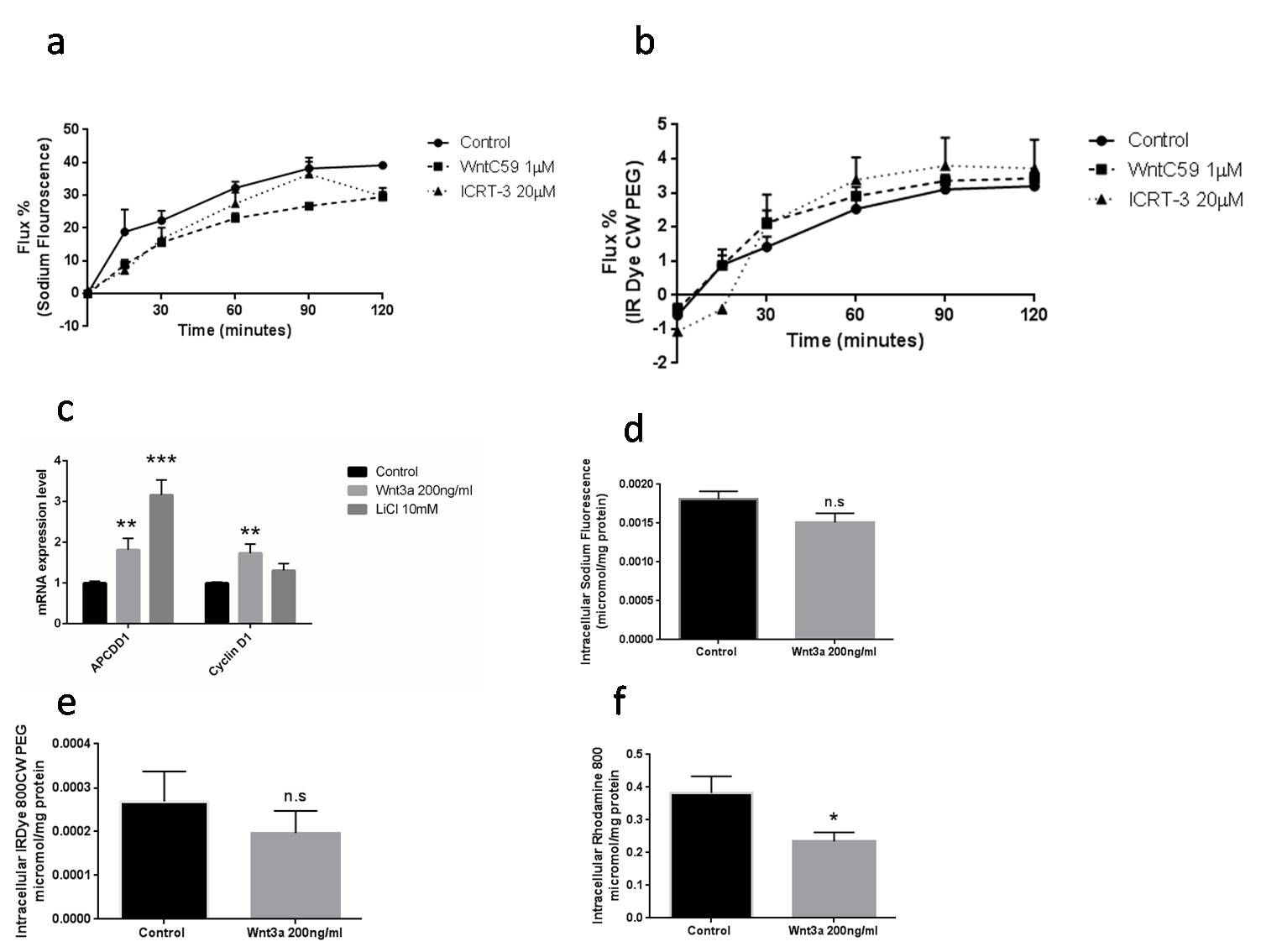


Figure S2: Permeability studies on Wnt inhibitior. a) Permeability of sodium fluorescein across hCMEC/D3 monolayer upon WntC59 or ICRT-3 treatment. b) Permeability of IR Dye 800CW PEG across hCMEC/D3 monolayer upon WntC59 or ICRT-3 treatment. c) Upregulation of APCDD1 and Cyclin D1 under Wnt3a and LiCl treatment. d, e and f) Intracellular accumulation of sodium fluorescein, IR Dye 800CW PEG and Rhodamine 800 post permeability study on cell that been treated with Wnt3a. a and b: n of 6-9, mean±SEM; c: n of 4, two way ANOVA followed by LSD Fisher’s test, d, e and f: One tail t-test, n of 3. *p<0.05; *p<0.01; ** p<0.001. mean±SEM.


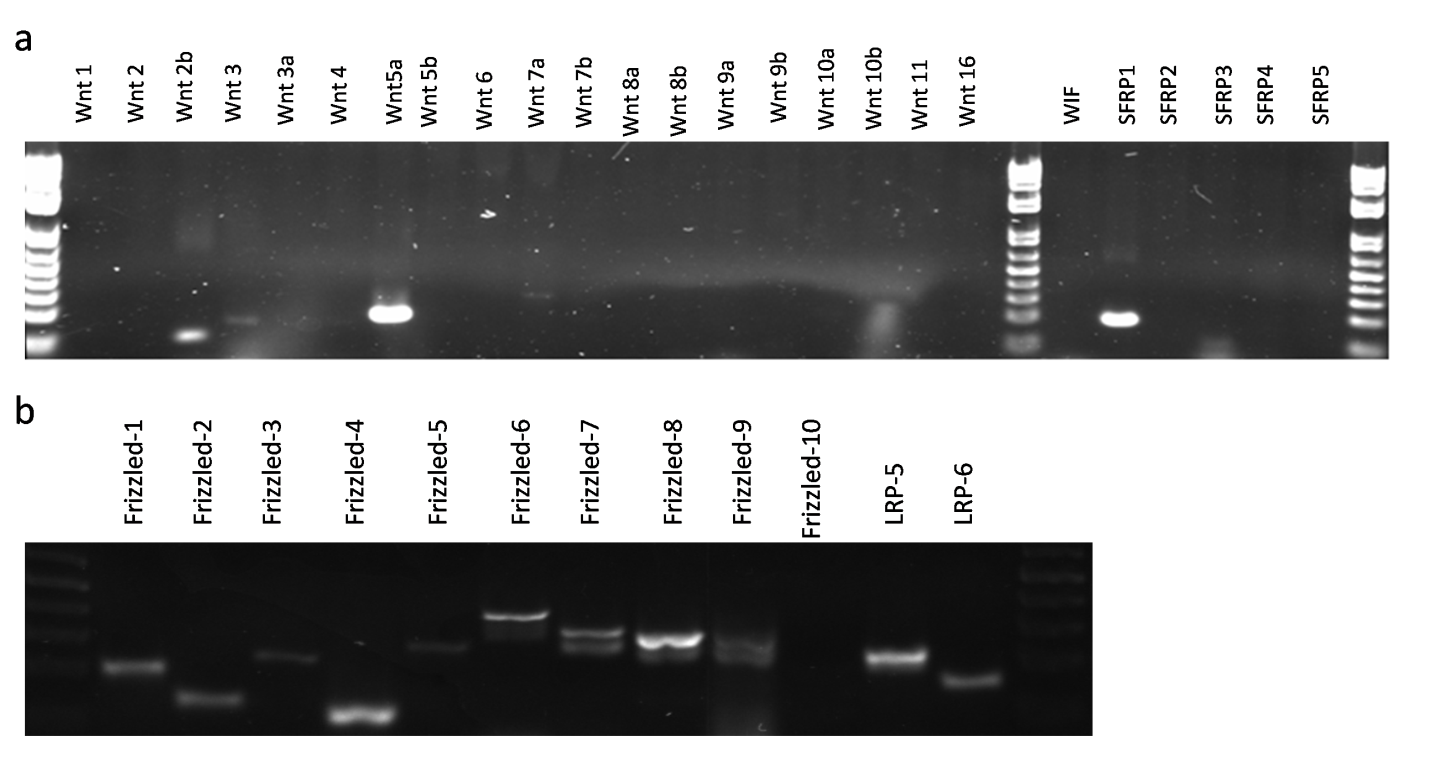


Figure S3: Expression of Wnt ligand, Wnt modulator (a) and Wnt receptor (b) at primary brain endothelial cell (HBMEC). HBMEC was cultured in completed EBM-2 media. Cell was harvested when reaching confluence.


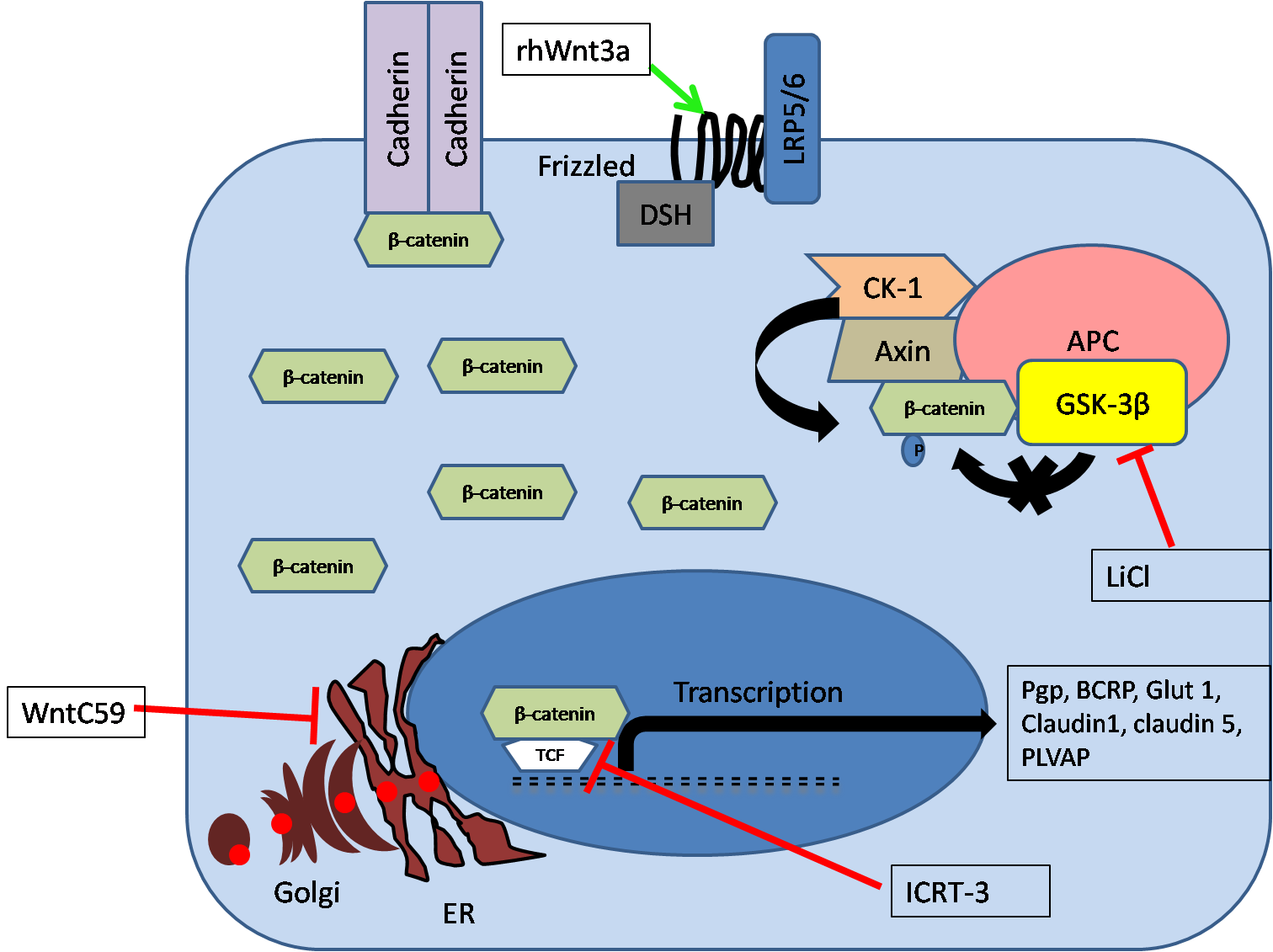


Figure S4: Modulation of Wnt/β-catenin signaling at immortalized human brain endothelial cell (hCMEC/D3). Both natural ligand (Wnt3a) and GSK inhibition (LiCl) activated Wnt/β-catenin signaling although Wnt3a produced more robust improvement in blood brain barrier function. Inhibition of Wnt released from HCMEC/D3 using WntC59 produced minimal changed on BBB function. More downstream Wnt inhibition by inhibiting β-catenin binding to TCF-4 using ICRT-3 resulted more dramatic changes in BBB function. Activation of Wnt/β-catenin upregulated P-glycoprotein, BCRP, claudin-1, claudin-5 and PLVAP.


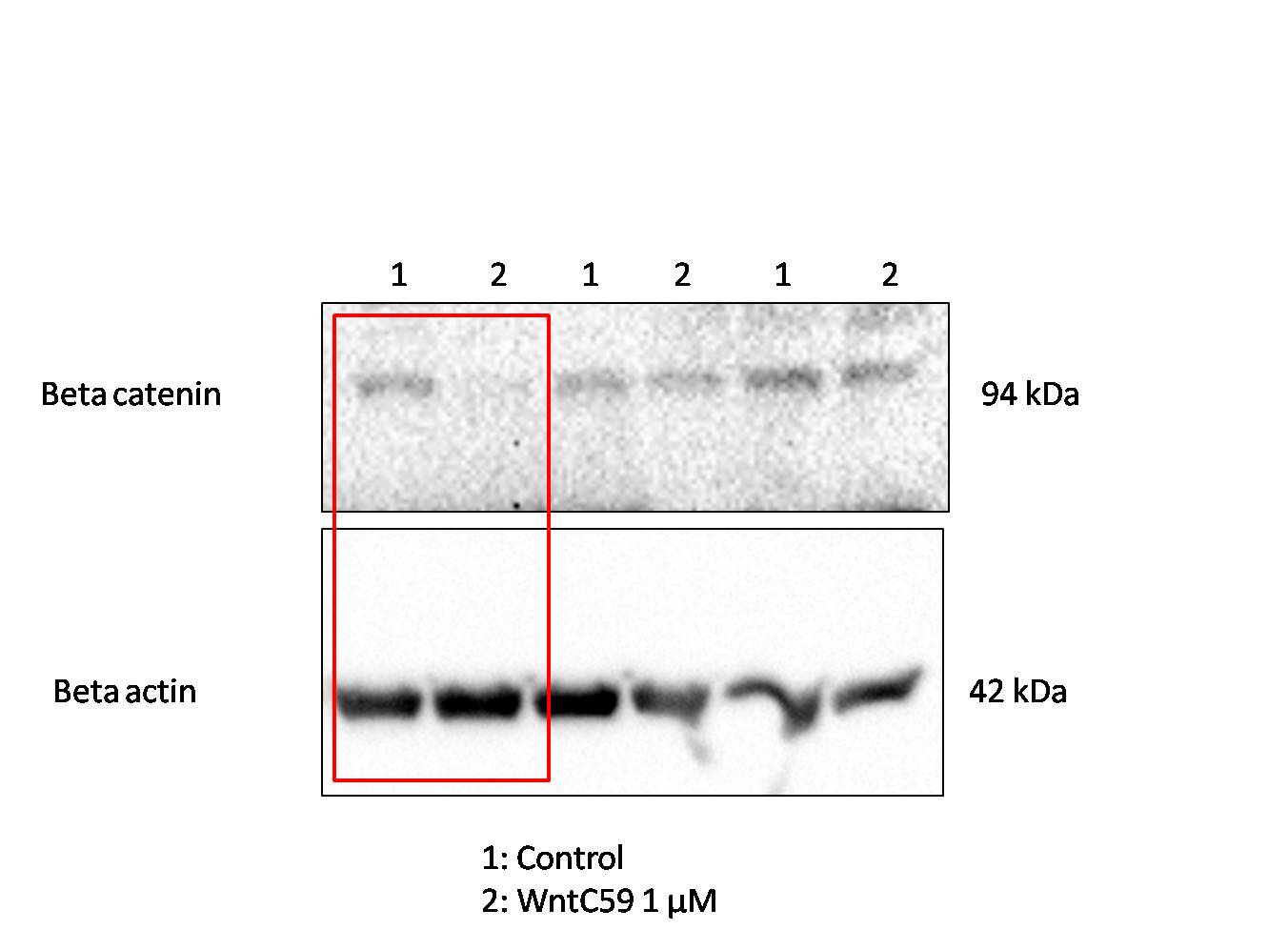


Figure S5: Original full lenght blot of figure 2a. Boxes indicated areas shown in the figure.


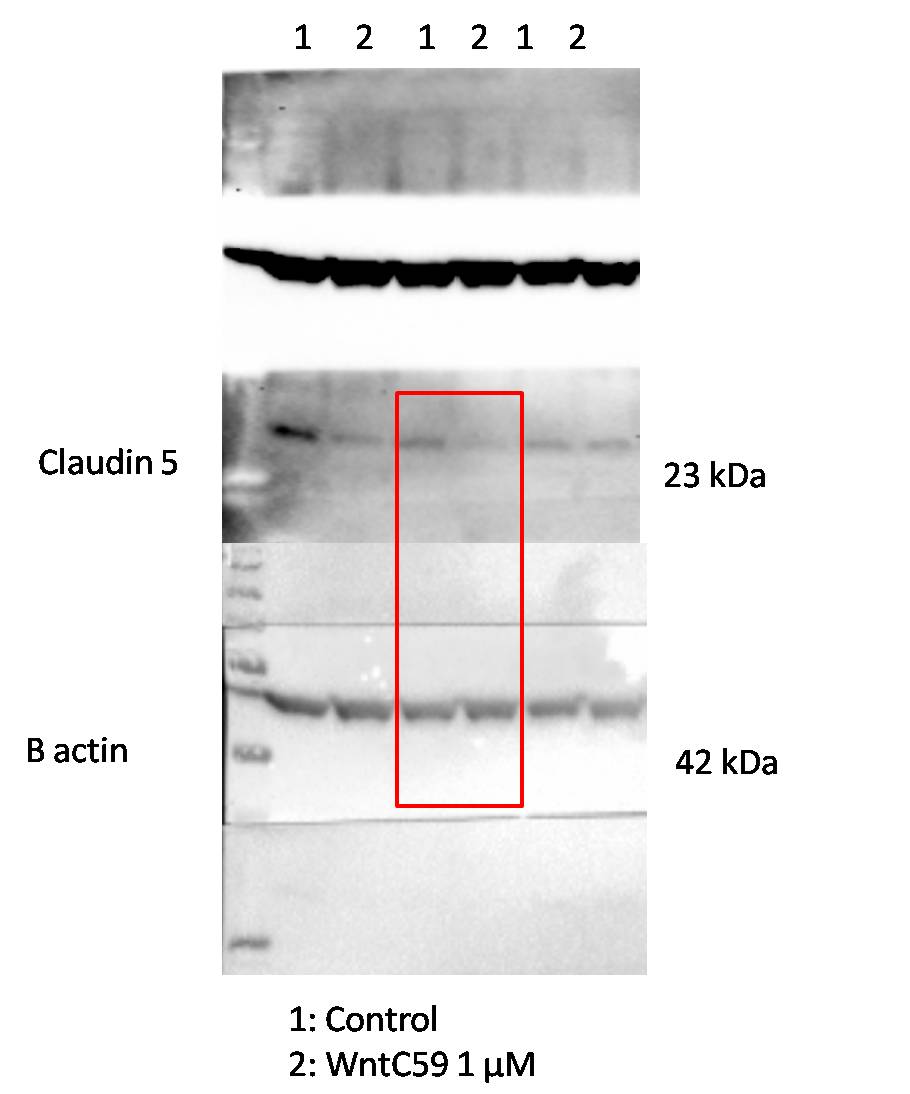


Figure S6: Original full lenght blot of figure 2d. Boxes indicated areas shown in the figure.


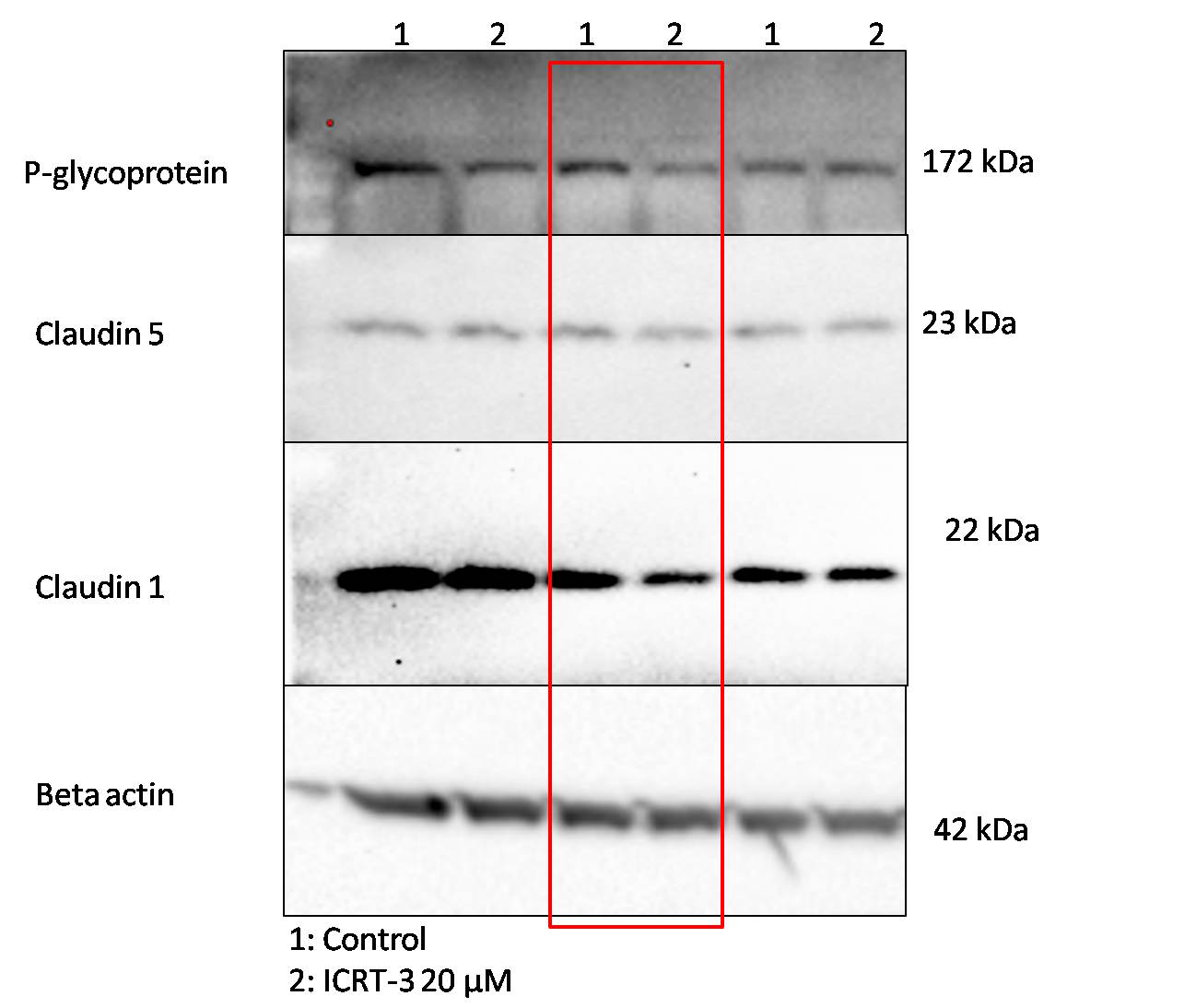


Figure S7: Original full lenght blot of figure 3b, 3c and 3d. Boxes indicated areas shown in the figure.


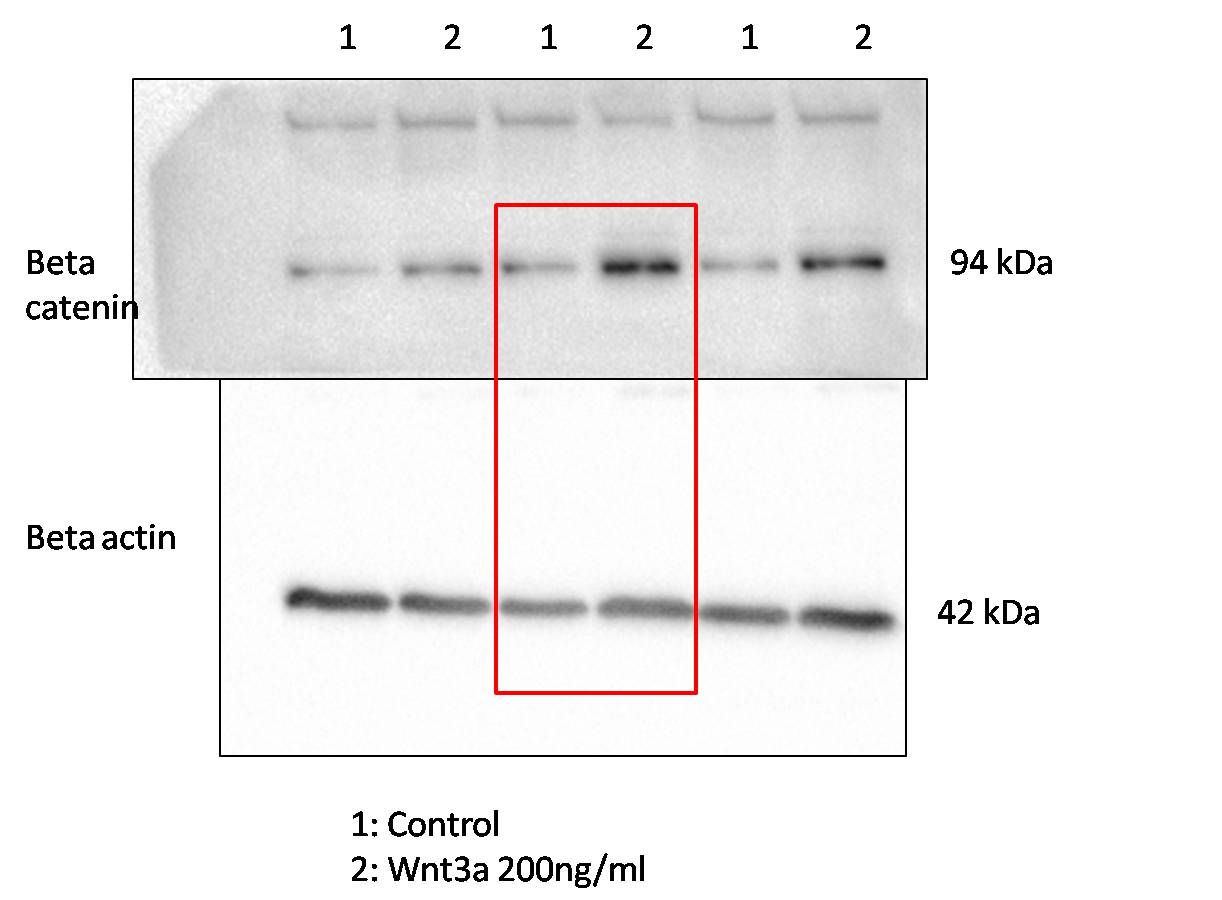


Figure S8: Original full lenght blot of figure 4a. Boxes indicated areas shown in the figure.


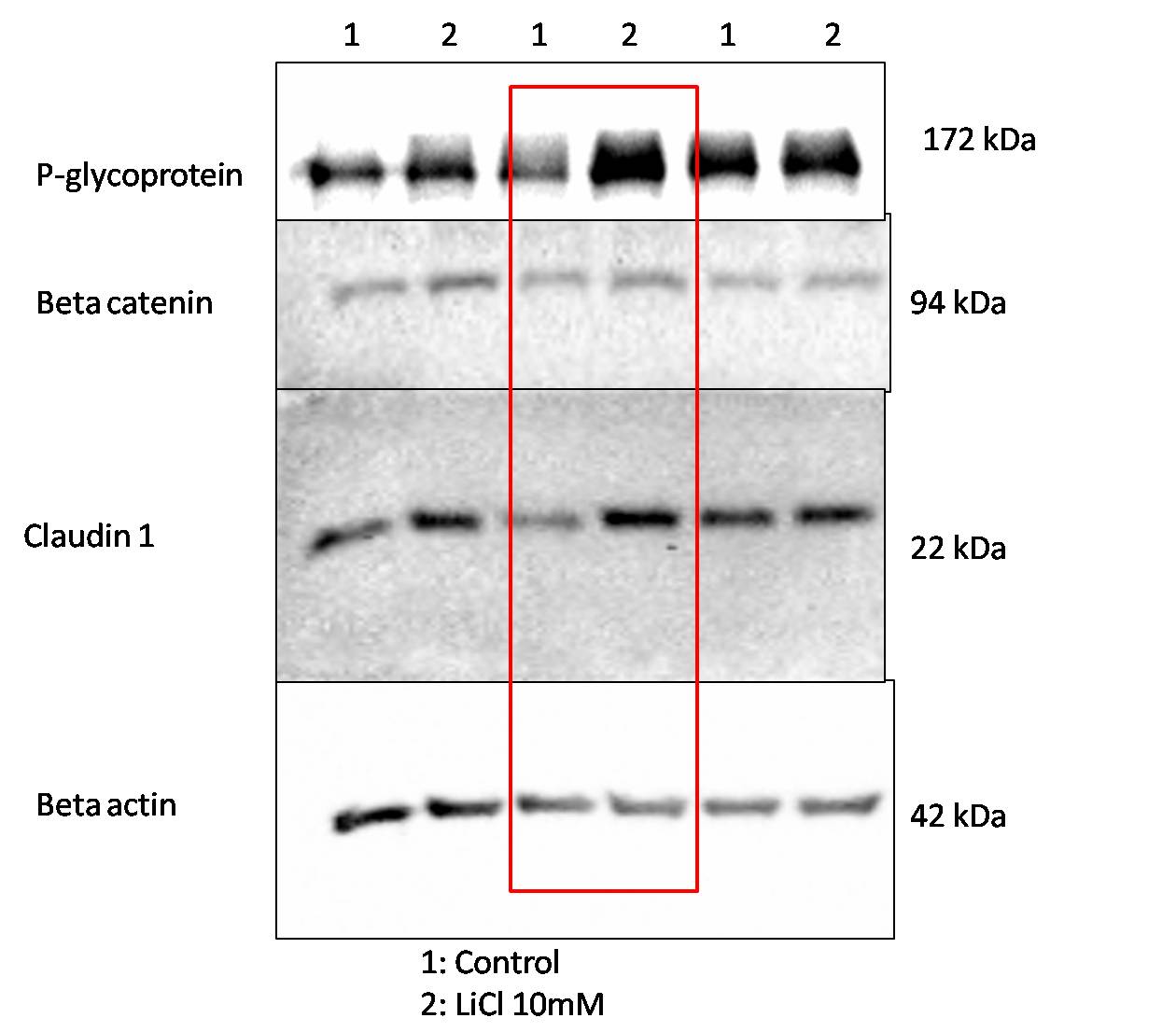


Figure S9: Original full lenght blot of figure 4b, 4f and 4g for LiCl. Boxes indicated areas shown in the figure.


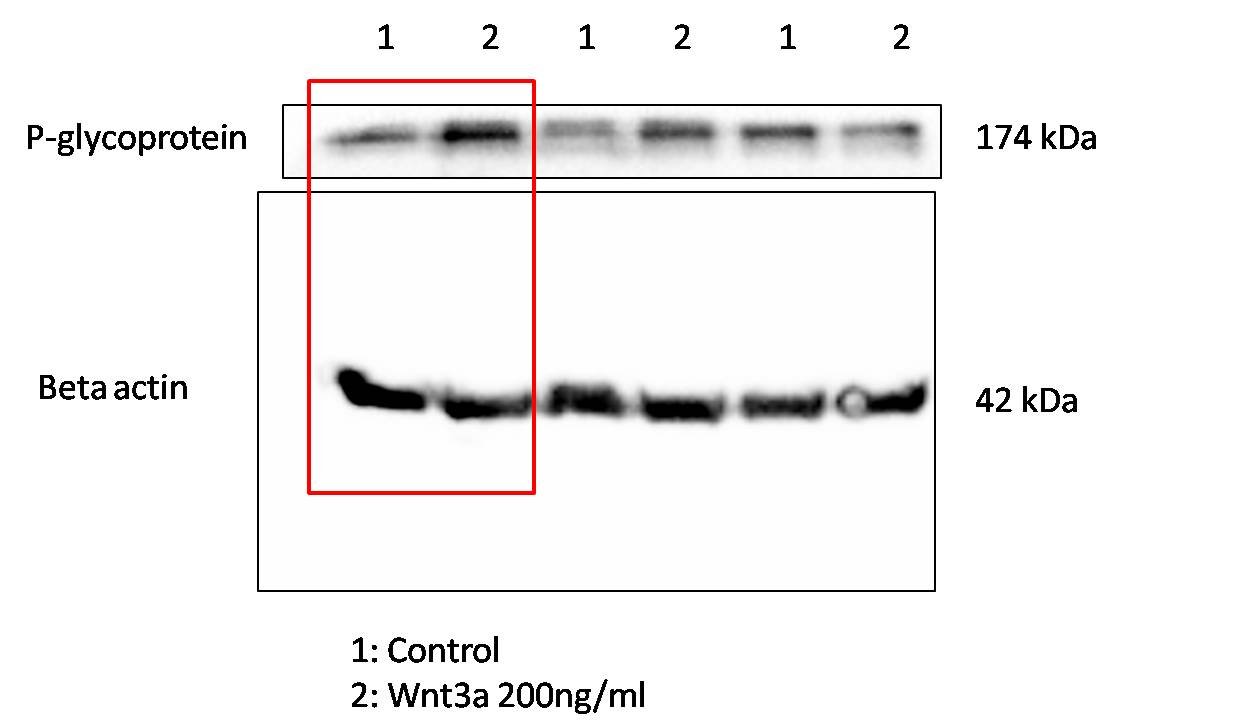


Figure S10: Original full lenght blot of figure 4f for Wnt3a. Boxes indicated areas shown in the figure.


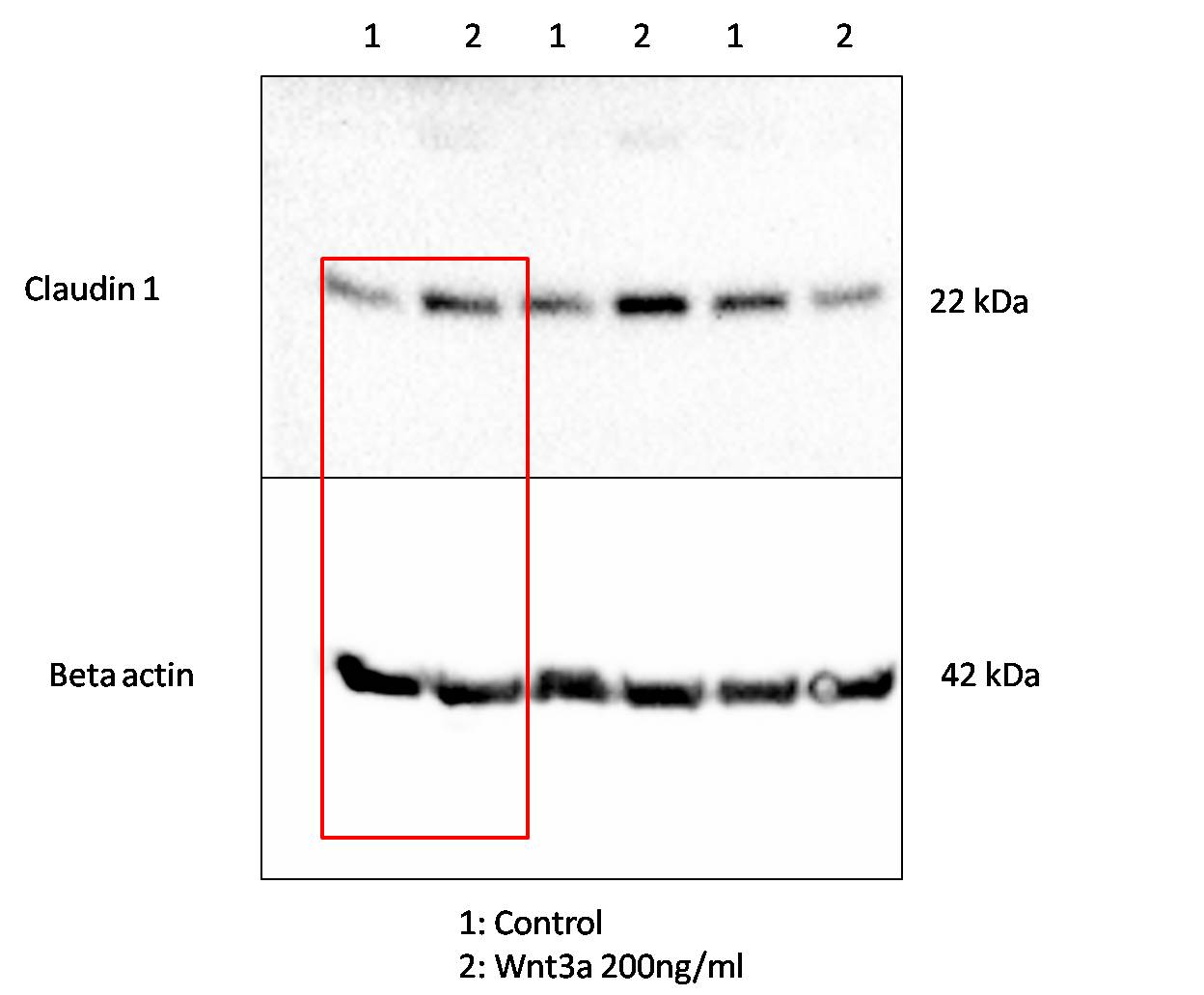


Figure S11: Original full lenght blot of figure 4g for Wnt3a. Boxes indicated areas shown in the figure.

Table 1. Primer sequence for Human Wnt machineries and BBB phenotypes.

| No | Primer | Sequence | |
| --- | --- | --- | --- |
| 1 | hWnt1-Forward | CAAGATCGTCAACCGAGGCT | |
| 2 | hWnt1-Reverse | TCACACGTGCAGGATTCGAT | |
| 3 | hWnt2-Forward | CGTGTGTGCAACCTGACTTC | |
| 4 | hWnt2-Reverse | TGTGTGCACATCCAGAGCTT | |
| 5 | hWnt2b-Forward | GATCCGAGAGTGTCAGCACC | |
| 6 | hWnt2b-Reverse | CCTCTCGGCTACTTCTGAGC | |
| 7 | hWnt3-Forward | TGACTCGCATCATAAGGGGC | |
| 8 | hWnt3-Reverse | GTGGTCCAGGATAGTCGTGC | |
| 9 | hWnt3a-Forward | AGCAGGACTCCCACCTAAAC | |
| 10 | hWnt3a-Reverse | AGAGGAGACACTAGCTCCAGG | |
| 11 | hWnt4-Forward | TCTTCGCCGTCTTCTCAGCC | |
| 12 | hWnt4-Reverse | GCACCGAGTCCATGACTTCC | |
| 13 | hWnt5a-Forward | TGTTGCTCGGCCCAGAAGTC | |
| 14 | hWnt5a-Reverse | GCTTCAATTACAACCTGGGCG | |
| 15 | hWnt5b-Forward | GCGAGAAGACTGGAATCAGGG | |
| 16 | hWnt5b-Reverse | TAATGACCACCAGGAGTTGGC | |
| 17 | hWnt6-Forward | CGGGGAGCGTTTAAAGGACA | |
| 18 | hWnt6-Reverse | TTATTGATACTAACCTCACCCACC | |
| 19 | hWnt7a-F-Forward | AGTACAACGAGGCCGTTCAC | |
| 20 | hWnt7a-Reverse | GCACGTGTTGCACTTGACAT | |
| 21 | hWnt7b-Forward | TACGTGAAGCTCGGAGCACT | |
| 22 | hWnt7b-Reverse | CGGAACTGGTACTGGCACTC | |
| 23 | hWnt8a-F-Forward | CTGGTCAGTGAACAATTTCC | |
| 24 | hWnt8a-Reverse | GTAGCACTTCTCAGCCTGTT |  |
| 25 | hWnt8b-Forward | TATCAGTTTGCCTGGGACCG | |
| 26 | hWnt8b-Reverse | CTGTCTCCCGATTGGCACTG | |
| 27 | hWnt10a-Forward | CTGTTCTTCCTACTGCTGCT | |
| 28 | hWnt10a-Reverse | ACACACACCTCCATCTGC | |
| 29 | hWnt10b-Forward | GTCTCCCCACGGTTTAAGCA | |
| 30 | hWnt10b-Reverse | TCAGGACCTCCAGTGGTTTG | |
| 31 | hWnt11-Forward | TCTTTGGGGTGGCACTTCTC | |
| 32 | hWnt11-Reverse | TCTGCCGAGTTCACTTGACG | |
| 33 | hWnt9a-Forward | GACGGTCAAGCAAGGATCTG | |
| 34 | hWnt9a-Reverse | TGCTCTCGCAGTTCTTCTCA | |
| 35 | hWnt9b-Forward | GTGTCTTGCCATAGCAGGCTT | |
| 36 | hWnt9b-Reverse | AATAAGGAGGCCGTGTGTCAG | |
| 37 | hWnt16-Forward | TCAGGGAGACCCTCTTCACAG | |
| 38 | hWnt16-Reverse | AGCAGGTACGGTTTCCTCTTG | |
| 39 | hFrizzled1-Forwad | GTGAGCCGACCAAGGTGTAT | |
| 40 | hFrizzled1-Reverse | CAGCCGGACAAGAAGATGAT | |
| 41 | hFrizzled2-Forward | GCGAAGCCCTCATGAACAAG | |
| 42 | hFrizzled2-Reverse | TCCGTCCTCGGAGTGGTTCT | |
| 43 | hFrizzled3-Forward | TGAGTGTTCGAAGCTCTATGG | |
| 44 | hFrizzled3-Reverse | ATCACGCACATGCAGAAAAG | |
| 45 | hFrizzled4-Forward | CAGTGAGGCATGGAGGTGTT | |
| 46 | hFrizzled4-Reverse | AAAGAGCTCAAGGGGCCATC | |
| 47 | hFrizzled5-Forward | TACCCAGCCTGTCGCTAAAC | |
| 48 | hFrizzled5-Reverse | AAAACCGTCCAAAGATAAACTGC | |
| 49 | hFrizzled6-Forward | TGGCCTGAGGAGCTTGAATGTGAC | |
| 50 | hFrizzled6-Reverse | TATCGCCCAGCAAAAATCCAATGA | |
| 51 | hFrizzled7-Forward | GTTTGGATGAAAAGATTTCAGGC | |
| 52 | hFrizzled7-Reverse | GACCACTGCTTGACAAGCACAC | |
| 53 | hFrizzled8-Forward | ACAGTGTTGATTGCTATTAGCATG | |
| 54 | hFrizzled8-Reverse | GTGAAATCTGTGTATCTGACTGC | |
| 55 | hFrizzled9-Forward | CCCTAGAGACAGCTGACTAGCAG | |
| 56 | hFrizzled9-Reverse | CGGGGGTTTATTCCAGTCACAGC | |
| 57 | hFrizzled10-Forward | ACACGTCCAACGCCAGCATG | |
| 58 | hFrizzled10-Reverse | ACGAGTCATGTTGTAGCCGATG | |
| 59 | hsFRP1-Forward | TGGCCCGAGATGCTTAAGTG | |
| 60 | hsFRP1-Reverse | CCTCAGTGCAAACTCGCTGG | |
| 61 | hsFRP2-Forward | CTCGCTGCTGCTGCTCTTC | |
| 62 | hsFRP2-Reverse | GGCTTCACATACCTTTGGAG | |
| 63 | hsFRP-3-Forward | ATGGTCTGCGGCAGCCCGG | |
| 64 | hsFRP-3-Reverse | CTGTCGTACACTGGCAGCTC | |
| 65 | hsFRP-4-Forward | GTTCCTCTCCATCCTAGTGG | |
| 66 | hsFRP-4-Reverse | GCTGAGATACGTTGCCAAAG | |
| 67 | hsFRP5-Forward | CTACTGGAGGGTGTTTTCAC | |
| 68 | hsFRP5-Reverse | CTTTCCCTTACCCTCTCCT | |
| 69 | hWIF1-Forward | CACCTGGATTCTATGGAGTG | |
| 70 | hWIF1-Reverse | ACAGAGGTCTCCCTGGTAAC | |
| 71 | hDKK-1-Forward | CAGGATTGTGTTGTGCTAGA | |
| 72 | hDKK1-Reverse | TGACAAGTGTGAAGCCTAGA | |
| 73 | hDKK-2-Forward | CTCAACTCCATCAAGTCCTC | |
| 74 | hDKK-2-Reverse | TACCTCCCAACTTCACACTC | |
| 75 | hDKK3-Forward | GAGGTTGAGGAACTGATGG | |
| 76 | hDKK3-Reverse | CCAGTCTGGTTGTTGGTTAT | |
| 77 | hDKK4-Forward | GTCCTGGACTTCAACAACAT | |
| 78 | hDKK4-Reverse | GTTGCATCTTCCATCGTAGT | |
| 79 | hPVLAP-Forward | CTGCGATGCCTTGCTCTTCAT | |
| 80 | hPVLAP-Reverse | AGTCCCTCCACAGGTTACGA | |
| 81 | hOccludin-Forward | AAG CAA GTG AAG GGA TCT GC | |
| 82 | hOccludin-Reverse | GGG GTT ATG GTC CAA AGT CA | |
| 83 | hGlut-1-Forward | AAT ACA CCA CCT CAC TCC TG | |
| 84 | hGut-1-Reverse | GAG GTA CGT GTA AGG GAC TG | |
| 85 | hBCRP-Forward | CAG TCT TCA AGG AGA TCA GC | |
| 86 | hBCRP-Reverse | CCA GTA CGA CTG TGA CAA TG | |
| 87 | hPgp-Forward | ATA TCA GCA GCC CAC ATC AT | |
| 88 | hPgp-Reverse | GAA GCA CTG GGA TGT CCG GT | |
| 89 | hVE-cadherin-Forward | GTT CGG CTG ACA GGT CCA CA | |
| 70 | hVE-cadherin-Reverse | CGA TGT GGC GAG GAG CAT CA | |
| 71 | hZO-1-Forward | ATC TCG GAA AAG TGC CAG GA | |
| 72 | hZO-1-Reverse | TTT CAG CGC ACC ATA CCA AC | |
| 73 | hClaudin-5-Forward | AGGCGTGCTCTACCTGTTTTG | |
| 74 | hClaudin-5-Reverse | AACTCGCGGACGACAATGTT | |
| 75 | hClaudin-3-Forw | GCCACCAAGGTCGTCTACTC | |
| 76 | hClaudin-3-Reverse | CGTAGTCCTTGCGGTCGTAG | |
| 77 | hClaudin-1-Forward | TTTACTCCTATGCCGGCGAC | |
| 78 | hClaudin-1-Reverse | GAGGATGCCAACCACCATCA | |
| 79 | hCyclin-D-Forward | GTCCCACTCCTACGATACGC | |
| 80 | hCyclin D-Reverse | CAGGGCCGTTGGGTAGAAAA | |
| 81 | hAPCDD1-Forward | AAGGAGTCACAGTGCCATCA | |
| 82 | hAPCDD1-Reverse | TTGTGATGAACTCTGGGCCT | |
| 83 | hAxIn-2-Forward | GACAGGAATCATTCGGCCAC | |
| 84 | hAxin-2-Reverse | CCTTCAGCATCCTCCGGTAT | |
